# Budding Yeast Dma Proteins Control Septin Dynamics and the Spindle Position Checkpoint by Promoting the Recruitment of the Elm1 Kinase to the Bud Neck

**DOI:** 10.1371/journal.pgen.1002670

**Published:** 2012-04-26

**Authors:** Laura Merlini, Roberta Fraschini, Barbara Boettcher, Yves Barral, Giovanna Lucchini, Simonetta Piatti

**Affiliations:** 1 Dipartimento di Biotecnologie e Bioscienze, Università di Milano-Bicocca, Milano, Italy; 2 Institute of Biochemistry, ETH Zurich, Zurich, Switzerland; 3 Centre de Recherche en Biochimie Macromoléculaire, Montpellier, France; The University of North Carolina at Chapel Hill, United States of America

## Abstract

The first step towards cytokinesis in budding yeast is the assembly of a septin ring at the future site of bud emergence. Integrity of this ring is crucial for cytokinesis, proper spindle positioning, and the spindle position checkpoint (SPOC). This checkpoint delays mitotic exit and cytokinesis as long as the anaphase spindle does not properly align with the division axis. SPOC signalling requires the Kin4 protein kinase and the Kin4-regulating Elm1 kinase, which also controls septin dynamics. Here, we show that the two redundant ubiquitin-ligases Dma1 and Dma2 control septin dynamics and the SPOC by promoting the efficient recruitment of Elm1 to the bud neck. Indeed, *dma1 dma2* mutant cells show reduced levels of Elm1 at the bud neck and Elm1-dependent activation of Kin4. Artificial recruitment of Elm1 to the bud neck of the same cells is sufficient to re-establish a normal septin ring, proper spindle positioning, and a proficient SPOC response in *dma1 dma2* cells. Altogether, our data indicate that septin dynamics and SPOC function are intimately linked and support the idea that integrity of the bud neck is crucial for SPOC signalling.

## Author Summary

During asymmetric cell division, spindle positioning is critical for ensuring the unequal inheritance of polarity factors. In budding yeast, the constriction between mother cell and bud, the bud neck, determines the cleavage plane. Cytoskeletal proteins called septins form a ring at the bud neck that serves as scaffold for the recruitment of proteins required for cytokinesis. The septin ring also interacts with cytoplasmic microtubules to orient the spindle along the mother–bud axis and to ensure that each daughter cell inherits one full set of chromosomes. A surveillance device called Spindle Position Checkpoint (SPOC) oversees this process and delays mitotic exit and cytokinesis until the spindle is properly oriented along the division axis, thus ensuring genome stability. Feedback mechanisms responding to spindle mispositioning likely exist also in higher eukaryotes. In this manuscript, we show that two budding yeast ubiquitin ligases are involved in the regulation of septin dynamics and the SPOC by promoting the bud neck localization of the Elm1 protein kinase, which in turn participates in both processes. Altogether our data indicate that bud neck integrity and septins are important for SPOC signalling and that the two processes are controlled by a common regulatory module.

## Introduction

How eukaryotic cells position their cleavage furrow for cytokinesis is a key question in cell biology. Cleavage furrow mispositioning eventually generates aneuploidies that can drive cells into tumorigenesis [1], [2]. Indeed, cytokinesis must be spatially and temporally coordinated with sister chromatid partition in order to generate cells with equal genetic information.

In many eukaryotic cells, cytokinesis is driven by a contractile actomyosin ring, which forms at the site of cell division and drives furrow ingression [3]. In budding yeast the first step towards cytokinesis is the assembly in late G1-phase of a rigid septin ring at the bud neck, the constriction between the mother cell and the bud that defines the future site of cleavage. The septin ring serves as a scaffold for the assembly of other proteins at the bud neck, such as components of the actomyosin ring [4]. In addition, once the septin ring splits in two during cytokinesis (see below), it generates a compartment where numerous membrane-remodelling proteins are confined for abscission [5].

The yeast septin ring forms at the site of bud emergence before expanding into a broader hourglass structure as the bud grows during S phase through mitosis. At cytokinesis onset, it splits into two separate rings [6]. Septin function is linked to the tight regulation of septin dynamics inside the ring [7], [8]. Septins associate dynamically within the ring during its formation in late G1 and its splitting at cytokinesis onset. This state is referred to as “fluid” state. However, septins stop moving as the ring turns into an hourglass-shaped collar at the bud neck, reaching its “frozen” state concomitantly with early bud emergence. This frozen state is maintained throughout bud growth during the S, G2 and M-phases. Several yeast kinases, such as Cla4, Gin4 [9] and Elm1 [10] locate at the bud neck in a septin-dependent manner and are involved in septin collar formation. Septin ring stabilization in S phase is promoted by phosphorylation events. In particular, Cla4 phosphorylates several septins [8], [11] and is regulated by Elm1 [12]. Elm1 also phosphorylates and activates Gin4, which in turn phosphorylates the Shs1 septin *in vitro* and *in vivo* [13], [14]. Moreover, Cla4 is involved in Gin4 recruitment to the bud neck and association with septins [14], [15], indicating another route through which Cla4 regulates septin collar stability.

At cytokinesis the scission of the septin collar into two separate rings correlates with Shs1 dephosphorylation and relocalization of protein phosphatase 2A bound to its B-type regulatory subunit Rts1 (PP2A^Rts1^) from kinetochores to the bud neck [8]. PP2A^Rts1^ appears to be a major activator of septin dynamics at cytokinesis, as its inactivation severely impairs this process [8].

The budding yeast septin ring is also involved in proper spindle positioning [16]. Indeed, the bud neck defines the position of cytokinesis long before spindle assembly, making proper spindle orientation along the mother-bud axis mandatory for chromosome segregation into the daughter cells. Whenever budding yeast cells experience spindle position defects, they undergo anaphase within the mother cell to then hold on in telophase with elongated spindles and high levels of mitotic CDKs until one of the daughter nuclei is properly segregated to the bud. This cell cycle delay is imposed by the spindle position checkpoint (SPOC), which responds to spindle position/orientation defects to avoid the generation of anucleate and binucleate cells [17], [18], [19]. The SPOC target is the Tem1 GTPase, whose active GTP-bound form promotes a signal transduction cascade called Mitotic Exit Network (MEN) that ultimately drives cells out of mitosis by leading to inactivation of mitotic CDKs [20], [21]. The dimeric GTPase-activating protein (GAP) Bub2/Bfa1 keeps Tem1 inhibited until the spindle is properly aligned, thus coupling mitotic exit with nuclear division [22], [23], [24]. The Kin4 protein kinase participates in the SPOC [25], [26] by keeping Bfa1 active [27] and by regulating the dynamics of Bub2/Bfa1 at spindle poles [28] (see below).

Tem1 and several downstream MEN components are found at SPBs in a cell cycle-regulated manner and are thought to promote mitotic exit from this location [29]. The Bub2/Bfa1 complex is found predominantly on the SPB that is pulled towards the bud, while it is present on both SPBs of misaligned spindles [24], [28], [30], [31], [32]. Kin4 is localized at the mother cell cortex, and accumulates at the bud neck in late anaphase. In mid-anaphase Kin4 also transiently appears on the mother-bound SPB, whereas it binds to both SPBs in case of spindle misalignment [25], [26]. Conversely, the Lte1 protein, which positively regulates Tem1, is confined in the bud from the G1/S transition to telophase, when it spreads throughout the cytoplasm of both mother cell and bud [22], [24]. Recent data suggest that Lte1 contributes to mitotic exit by preventing Kin4 from binding to the daughter-directed SPB [33], [34]. In addition, Lte1 regulates the loss of Bfa1 from the maternal SPB [35]. Spreading of Lte1 into the mother cell by *LTE1* overexpression or septin defects overcomes the SPOC-induced mitotic arrest [22], [36].

Integrity of the bud neck is thought to be important for SPOC signalling. Indeed, mutations interfering with the septin ring lead to improper mitotic exit in the presence of mispositioned spindles [36] and alter the residence time of Bub2/Bfa1 at the mother SPB [37]. In addition, the bud neck-localized Elm1 kinase [38], [39] and the PP2A^Rts1^ phosphatase [38], [40], which are involved in regulation of septin ring stability and dynamics [10], [12], [41], contribute to the SPOC by regulating Kin4 kinase activity and localization, respectively.

We previously implicated the redundant ubiquitin-ligases Dma1 and Dma2 in septin ring integrity, spindle positioning, cytokinesis and SPOC regulation [42]. Dma1 and Dma2 share 58% identity in their primary sequence and contain a central FHA domain, usually involved in binding Thr-phosphorylated proteins [43], as well as a C-terminal RING finger domain, typical of E3 ubiquitin ligases [44]. Here we extend our previous observations by showing that Dma proteins contribute to the stabilization of the septin ring throughout the cell cycle and are implicated in the SPOC by regulating the bud neck localization of the Elm1 kinase. Impaired recruitment of Elm1 to the bud neck in *dma1Δ dma2Δ* mutant cells results in decreased Elm1-dependent phosphorylation of Kin4 on T209 and in misregulated localization of the Bub2/Bfa1 complex at the poles of mispositioned spindles without affecting Elm1 kinase activity. Strikingly, constitutive recruitment of Elm1 at the bud neck through a chimeric protein is sufficient to rescue the septin defects and the SPOC failure of *dma1Δ dma2Δ* cells, indicating that the checkpoint defect in these cells is indeed due to loose Elm1 association with the bud neck. Thus, altogether our data strongly indicate that septin dynamics and SPOC response are interconnected and provide the first mechanistic insight into the role of the Dma ubiquitin ligases in these processes.

## Results

### Dma proteins control septin ring deposition and maintenance

We previously showed that simultaneous lack of Dma1 and Dma2 causes cell lethality in the absence of the PAK kinase Cla4. Moreover, a *dma1Δ dma2Δ cla4-75* conditional mutant exhibits severe defects in bud neck morphology and septin ring localization under restrictive conditions, indicating that Dma proteins might be involved in septin dynamics [42].

To further investigate this issue, we analysed the consequences of deleting *DMA1* and *DMA2* in septin mutants, such as *cdc12-1*, *cdc12-6* and *shs1Δ*, and in mutants lacking protein kinases that regulate septin dynamics, such as *kcc4Δ*, *gin4Δ*, *elm1Δ* and *hsl1Δ* [45]. Consistent with our previous data, lack of Dma proteins caused cell lethality or severe growth defects when combined with conditional septin mutations or with *GIN4*, *HSL1* and *ELM1* deletion (Table 1), supporting the notion that Dma1 and Dma2 might regulate septin ring dynamics. Conversely, they were not essential in the absence of the Kcc4 protein kinase, which was shown to play a minor role in this process [15].

**Table 1:** Synthetic effects between the lack of Dma proteins and mutations affecting septin dynamics.

GENOTYPE	PHENOTYPE AT 25°C
*dma1Δ dma2Δ kcc4Δ*	HEALTHY
*dma1Δ dma2Δ hsl1Δ*	LETHAL/VERY SICK
*dma1Δ dma2Δ gin4Δ*	LETHAL/VERY SICK
*dma1Δ dma2Δ elm1Δ*	LETHAL/VERY SICK
*dma1Δ dma2Δ cla4Δ*	LETHAL
*dma1Δ dma2Δ cdc12-1*	LETHAL/VERY SICK
*dma1Δ dma2Δ cdc12-6*	LETHAL/VERY SICK
*dma1Δ dma2Δ shs1Δ*	LETHAL/VERY SICK

Strains carrying *DMA1* and *DMA2* deletions were crossed with mutants defective in septin dynamics. The resulting diploids were induced to sporulate and meiotic segregants.

**Figure 1:**
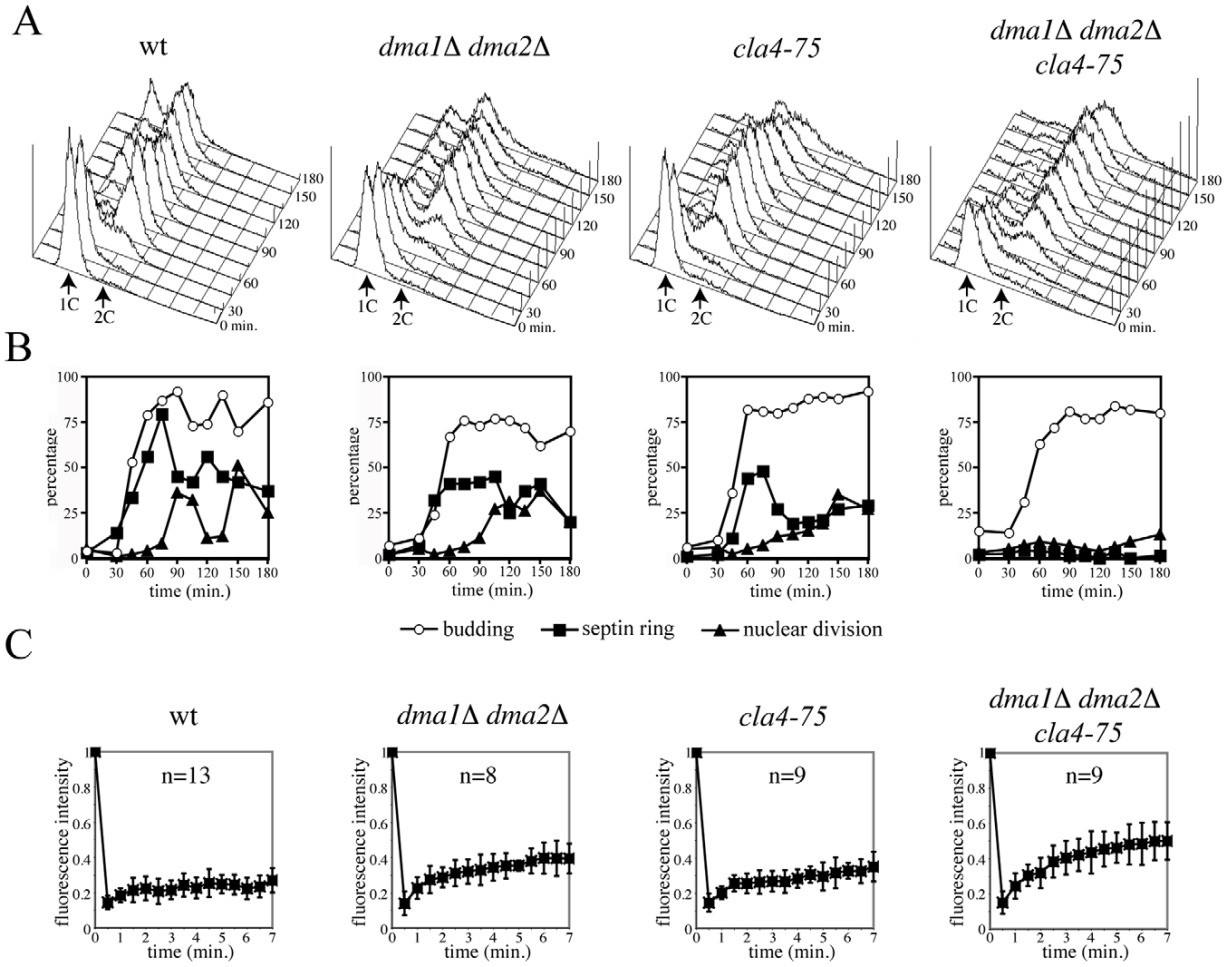
Dma proteins are required together with Cla4 for septin ring deposition. A–B: Logarithmically growing cultures of wild type (ySP3070), *dma1Δ dma2Δ* (ySP4319), *cla4-75* (ySP4320) and *dma1Δ dma2Δ cla4-75* (ySP4321) cells, all expressing a fusion of the Cdc3 septin with GFP, were arrested in G1 by alpha factor at 25°C and released into the cell cycle at 37°C. At the indicated times, cell samples were taken for FACS analysis of DNA contents (A) and for scoring budding, nuclear division and septin ring deposition (B). C: Septin rings labelled by GFP-Cdc12 were subjected to FRAP using the following strains: ySP8176 (wt), ySP8173 (*dma1Δ dma2Δ*), ySP8296 (*cla4-75*) and ySP8193 (*dma1Δ dma2Δ cla4-75*). Half of the ring was bleached at time 0. Recovery of fluorescence was measured over time upon shift to 37°C and plotted as fraction of fluorescence intensity relative to time 0 (unbleached). Bars indicate standard errors.

We then asked if Dma proteins control assembly and/or maintenance of the septin ring. Septin ring assembly was analysed during a synchronous release from G1 of *dma1Δ dma2Δ cla4-75* conditional mutant cells [42]. Cultures of wild type, *dma1Δ dma2Δ*, *cla4-75* and *dma1Δ dma2Δ cla4-75* cells, all expressing a *CDC3-GFP* fusion to visualize the septin ring, were arrested in G1 by alpha factor at 25°C and released in the cell cycle at 37°C to inactivate the temperature-sensitive *cla4-75* allele. Samples were taken at different time points to monitor kinetics of DNA replication (Figure 1A), budding, nuclear division and septin ring assembly (Figure 1B). Both *dma1Δ dma2Δ* and *cla4-75* cells underwent proper deposition of the septin ring with kinetics similar to wild type, although with somewhat reduced efficiency. Conversely, *dma1Δ dma2Δ cla4-75* cells were virtually unable to assemble the septin ring at the bud neck throughout the duration of the experiment, suggesting that Dma proteins and Cla4 share overlapping function(s) in septin ring deposition and/or stabilization. In addition, *dma1Δ dma2Δ cla4-75* cells did not undergo nuclear division and arrested in G2 as budded, mononucleated cells.

To assess whether Dma1 and Dma2, together with Cla4, control the stability of the septin ring, we analysed the latter in *dma1Δ dma2Δ cla4-75* cells that were shifted to the restrictive temperature at various cell cycle stages after elutriation of small unbudded cells. Whereas the septin ring was fairly stable in wild type cells after shift to 37°C, it rapidly mispositioned and eventually disassembled in *dma1Δ dma2Δ cla4-75* cells at the restrictive temperature, irrespective of the cell cycle stage at which cells were shifted to 37°C (Figure S1A, S1B). Similarly, in cells arrested in mitosis by nocodazole treatment and then shifted to 37°C, the septin ring progressively disassembled in *dma1Δ dma2Δ cla4-75* cells (Figure S1C). The role of Dma proteins in septin ring deposition and stabilization requires their FHA and RING domains (Figure S2A, S2B), suggesting that it depends on their E3 ubiquitin-ligase activity.

We next investigated by fluorescence recovery after photobleaching (FRAP) [8] whether Dma proteins and Cla4 inactivation would affect septin dynamics. Half of the fluorescently-labelled septin ring of cells expressing GFP-tagged Cdc12 was irradiated with a laser beam to irreversibly bleach GFP in large budded cells, i.e. when the septin ring is normally in the frozen state. Consistently, fluorescence recovery on the ring was negligible in wild type cells at 37°C, indicating that septin complexes are immobile inside the ring. In contrast, inactivation of Dma proteins and Cla4 increased recovery in an additive way (Figure 1C), as indicated by the recruitment of fluorescent molecules to the bleached area. Thus, Dma proteins control septin ring dynamics throughout the cell cycle, independently of Cla4, and promote the establishment and/or maintenance of the frozen state of the septin ring.

### Deletion of *RTS1* suppresses the lethality and septin defects of *dma1 dma2 cla4* mutants

**Figure 2:**
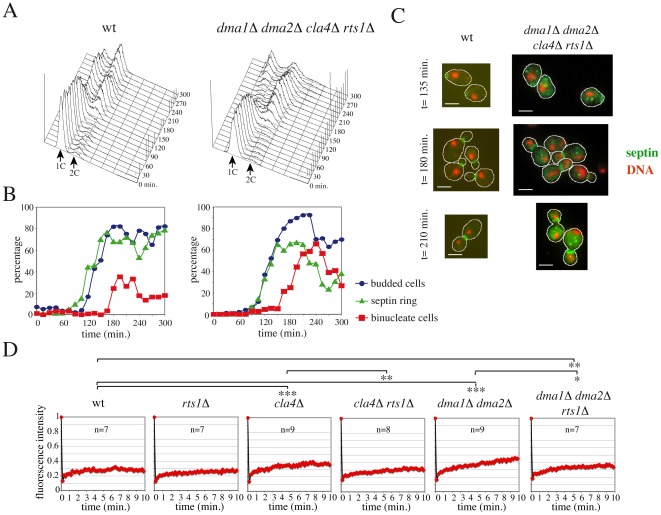
*RTS1* deletion rescues septin defects of *dma1Δ dma2Δ cla4Δ* cells. A–C: Small unbudded cells of wild type (W303) and *dma1Δ dma2Δ cla4Δ rts1Δ* (ySP8679) strains were isolated by centrifugal elutriation and resuspended in fresh medium at 25°C at time 0. At the indicated times, cell samples were taken for FACS analysis of DNA contents (A), for scoring budding and nuclear division and for in situ immunofluorescence of the septin ring with anti-Cdc11 antibodies (B). Micrographs of septin rings in representative wild type and *dma1Δ dma2Δ cla4Δ rts1Δ* cells are shown in (C). Scale bars: 5 µm. D: Septin rings labelled by GFP-Cdc12 were subjected to FRAP using the following strains: ySP8176 (wt), ySP8173 (*dma1Δ dma2Δ*), ySP8701 (*rts1Δ*), ySP8703 (*dma1Δ dma2Δ rts1Δ*), ySP8280 (*cla4Δ*) and ySP9476 (*cla4Δ rts1Δ*). Half of the ring was bleached at time 0. Recovery of fluorescence was measured over time (10 seconds intervals) at 25°C and plotted as fraction of fluorescence intensity relative to time 0 (unbleached). *:p<0.05; **:p<0.01; ***:p<0.001, *t*-test.

PP2A^Rts1^ promotes septin ring destabilization during ring splitting and prior to cytokinesis probably by direct septin dephosphorylation [8]. We therefore asked if *RTS1* deletion could rescue the septin defects of *dma1 dma2 cla4* mutants. Lack of *RTS1* caused temperature-sensitive growth defects by itself and therefore did not suppress the temperature-sensitivity of *dma1Δ dma2Δ cla4-75* cells, but it rescued the lethality of *dma1Δ dma2Δ cla4Δ* cells at 25°C (data not shown). To analyse the kinetics of septin ring deposition and disassembly during the cell cycle, small unbudded wild type and *dma1Δ dma2Δ cla4Δ rts1Δ* cells were isolated by elutriation and then released in fresh medium at 25°C. Cell samples were then withdrawn at different time points for FACS analysis of DNA contents, kinetics of budding and nuclear division, as well as septin ring staining by in situ immunofluorescence. A septin ring was efficiently assembled in *dma1Δ dma2Δ cla4Δ rts1Δ* cells, albeit with a slight delay relative to budding and to the kinetics of septin deposition observed in wild type cells (Figure 2B, 2C). Accordingly, *dma1Δ dma2Δ cla4Δ rts1Δ* cells proceeded through mitosis and ultimately divided, giving rise to unbudded daughter cells with 1C DNA contents (Figure 2A, 2B). It should be noted, however, that *dma1Δ dma2Δ cla4Δ rts1Δ* cells grew more slowly (data not shown) and showed a higher septin cytoplasmic signal than wild type cells (Figure 2C), suggesting that their septin ring might not be as rigid as in wild type.

In order to understand whether lack of PP2A^Rts1^ suppresses the septin defects of *cla4* and/or *dma1 dma2* mutants, we assessed septin ring stability by FRAP. Deletion of *RTS1* significantly stabilized the septin ring of both *cla4Δ* and *dma1Δ dma2Δ* cells, although the septin ring remained more dynamic in *dma1Δ dma2Δ rts1Δ* than in wild type cells (Figure 2D).

Hydroxyurea treatment magnifies the septin defects of *dma1Δ dma2Δ* cells, leading to a significant percentage of aberrantly shaped cells with mislocalized septins ([46] and Figure S3A). *RTS1* deletion rescued the septin defects of *dma1Δ dma2Δ* cells also in these conditions (Figure S3A). Collectively, these data indicate that PP2A^Rts1^ acts antagonistically to Dma1, Dma2 and Cla4 in the regulation of septin dynamics. However, whereas *RTS1* deletion efficiently suppressed the morphological defects of *cla4Δ* cells (data not shown), consistent with previous data [47], *dma1Δ dma2Δ rts1Δ* mutants were quite unhealthy, as they grew more slowly and had a lower maximal permissive temperature than *dma1Δ dma2Δ* or *rts1Δ* cells (Figure S3B).

### The lethal effects of *DMA2* overexpression are suppressed by septin ring destabilization

**Figure 3:**
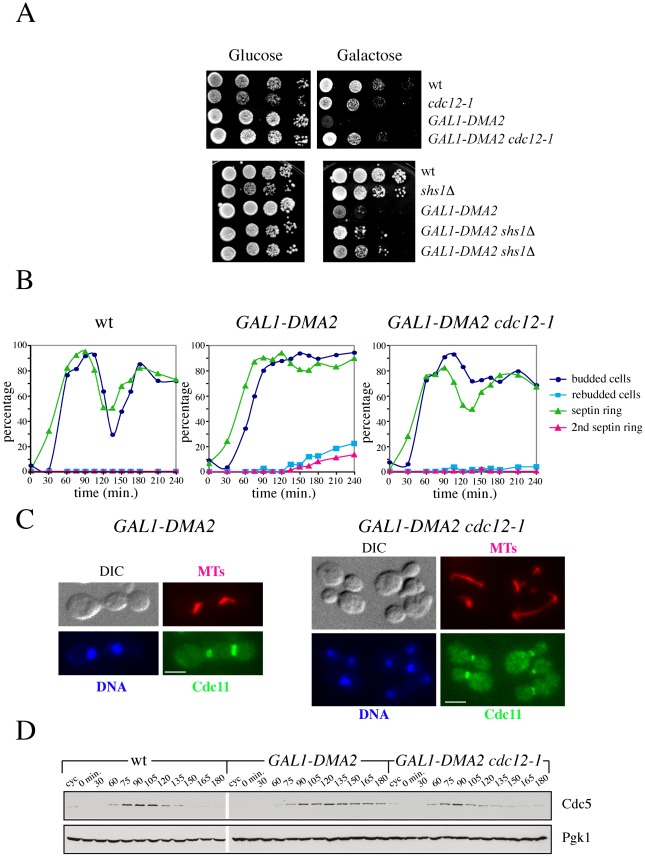
Destabilization of the septin ring rescues the lethality and cytokinesis defects caused by high levels of Dma2. A: Serial dilutions of stationary phase cultures of strains with the indicated genotypes were spotted on YEPD (glucose) or YEPRG (galactose) plates and incubated at 25°C for 2 days. B–D: Wild type (W303), *GAL1-DMA2* (ySP3018) and *GAL1-DMA2 cdc12-1* (ySP5244) cells grown in YEPR medium were arrested in G1 by alpha factor and released in YEPRG at 25°C. Alpha factor (10 µg/ml) was re-added at t = 105′ to the aliquot of culture used to make protein extracts (D), to prevent cells from entering a second cell cycle. At the indicated times, samples were taken for FACS analysis of DNA contents (not shown) and for scoring budding, nuclear division, spindle formation and septin ring deposition by *in situ* immunofluorescence with anti-tubulin and anti-Cdc11 antibodies, respectively (B). Representative micrographs were taken at t = 210′ (C). Scale bars: 5 µm. Protein extracts were analysed by western blot with anti-Cdc5 and anti-Pgk1 (loading control) antibodies (D).

Yeast cells carrying multiple copies of a *GAL1-DMA2* construct integrated in the genome show a pronounced cytokinesis defect when shifted to galactose-containing medium. In particular, these cells delay septin ring splitting but nevertheless enter a new cell cycle with assembly of a new septin ring at the site of bud emergence (Figure S4 and [42]). We asked if the cytokinesis defects caused by high levels of Dma2 are due to excessive stabilization of the septin ring by testing whether mutations that destabilize the septin ring, such as *cdc12-1* and *shs1Δ*, could rescue the lethality and restore normal septin dynamics in *DMA2*-overexpressing cells. Indeed, *GAL1-DMA2* toxic effects on galactose plates were partially suppressed by both *cdc12-1* and *shs1Δ* alleles at 25°C (Figure 3A), suggesting that septin ring destabilization can allow *GAL1-DMA2* cells to undergo proper cytokinesis. Septin ring formation and disassembly were then directly analyzed in *GAL1-DMA2* and *GAL1-DMA2 cdc12-1* cells during a synchronous release from G1 in galactose-containing medium at 25°C. In parallel, we monitored other cell cycle parameters, such as spindle assembly/disassembly and the levels of the polo kinase Cdc5, which is degraded during mitotic exit and we previously showed to be stabilized in *DMA2*-overexpressing cells [42]. *GAL1-DMA2* cells displayed cytokinesis defects, as shown by the failure to re-accumulate unbudded cells at the end of the cell cycle (Figure 3B), and were delayed in Cdc5 proteolysis (Figure 3D). As expected, a fraction (15–20%) of *GAL1-DMA2* cells rebudded and formed new septin rings before dividing (Figure 3B, 3C). In sharp contrast, such cytokinesis defects were suppressed in *GAL1-DMA2 cdc12-1* cells, which also displayed wild type kinetics of Cdc5 degradation. Altogether, these data support the notion that high levels of Dma2 cause septin ring hyperstabilization, which in turn likely causes cytokinesis failure and impairs timely degradation of Cdc5.

### Simultaneous lack of Dma1, Dma2, Cla4, and Swe1 causes spindle mispositioning without activation of the spindle position checkpoint

**Figure 4:**
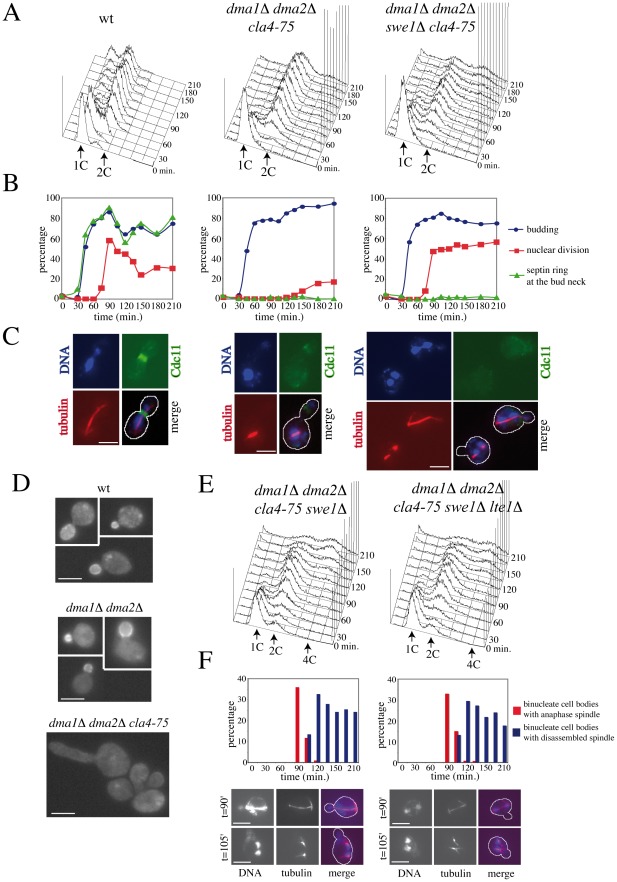
Morphogenesis checkpoint and SPOC response to spindle mispositioning in cells lacking Dma proteins and Cla4. A–C: Logarithmically growing cultures of wild type (W303), *dma1Δ dma2Δ cla4-75* (ySP5247) and *dma1Δ dma2Δ cla4-75 swe1Δ* (ySP6238) cells were arrested in G1 by alpha factor at 25°C and released into the cell cycle at 37°C at time 0. Cells were collected at the indicated times for FACS analysis of DNA contents (A) and scoring of budding, nuclear division, spindle assembly and septin ring assembly by immunofluorescence with anti-tubulin and anti-Cdc11 antibodies, respectively (B). Representative micrographs were taken 90 minutes after release (C). Scale bars: 5 µm. D: Localization of Lte1-GFP in wild type (ySP3333), *dma1Δ dma2Δ* (ySP4380) and *dma1Δ dma2Δ cla4-75* (ySP7766) cells expressing Lte1-GFP from the *GAL1* promoter after induction with 1% galactose for 1 hour followed by shift to 37°C for 3 hours. Scale bars: 5 µm. E–F: Cell cultures of *dma1Δ dma2Δ cla4-75 swe1Δ* (ySP6238) and *dma1Δ dma2Δ cla4-75 swe1Δ lte1Δ* (ySP7672) cells were treated as above. E: FACS analysis of DNA contents. F: the percentage of cells undergoing mispositioned anaphase (i.e. binucleate cell bodies with or without an intact anaphase spindle) was calculated over the total amount of binucleate cells. Nuclei were stained with DAPI and spindles were visualized by immunostaining of tubulin. Micrographs show representative cells undergoing anaphase in the mother cell and with an intact (t = 90′) or a disassembled spindle (t = 105′). Scale bars: 5 µm.

Because *dma1Δ dma2Δ cla4-75* cells are unable to deposit proper septin rings, their G2 arrest is likely due to activation of the morphogenesis checkpoint that responds to septin defects by delaying mitotic entry [45], [48]. We tested this hypothesis by knocking-out the direct target of this checkpoint, Swe1 [49], in cells lacking Dma1, Dma2 and Cla4. Wild type, *dma1Δ dma2Δ cla4-75* and *dma1Δ dma2Δ cla4-75 swe1Δ* cells were arrested in G1 by alpha factor at 25°C and then released into the cell cycle at 37°C. As expected, deletion of *SWE1* allowed *dma1Δ dma2Δ cla4-75* cells to undergo nuclear division at the restrictive temperature (Figure 4B, 4C), indicating that septin defects activate the morphogenesis checkpoint in these cells. Interestingly, nuclear division took place in the mother cell in a significant fraction of *dma1Δ dma2Δ cla4-75 swe1Δ* cells (35% of the anaphase cells at 90 minutes), which is consistent with the known role of the septin ring in spindle positioning [16]. In spite of that, cells exited mitosis and underwent a new round of DNA replication (Figure 4A), suggesting that the spindle position checkpoint, which normally prevents mitotic exit and cytokinesis in cells with mispositioned spindles, is inactive in these cells. The unscheduled mitotic exit observed under these conditions is unlikely caused by lack of Swe1, since *swe1Δ* cells are SPOC-proficient (see below, Figure S5A).

### SPOC proteins are mislocalized in *dma1Δ dma2Δ cla4-75 swe1Δ* cells

As septins and Cla4 are required to restrict Lte1 localization to the bud [36], [50], [51], [52], we wondered whether Lte1 spreading into the mother cell caused unscheduled mitotic exit in *dma1Δ dma2Δ cla4-75* cells. Indeed, Lte1 was homogeneously distributed in *dma1Δ dma2Δ cla4-75* cells in all cell cycle stages (Figure 4D). However, *LTE1* deletion did not affect the ability of *dma1Δ dma2Δ cla4-75 swe1Δ* cells to exit mitosis upon spindle mispositioning, as shown by the timely spindle disassembly in cells that underwent nuclear division in the mother compartment (Figure 4F), and enter into a new round of DNA replication (Figure 4E), suggesting that Dma proteins might control mitotic exit in ways that do not involve Lte1 localization/activation. Consistent with this conclusion, Lte1 localization at the bud cortex was not affected by the lack of Dma proteins (Figure S5B) and the SPOC defect of *dma1Δ dma2Δ* cells was not suppressed by *LTE1* deletion (see below, Figure S5C).

**Figure 5:**
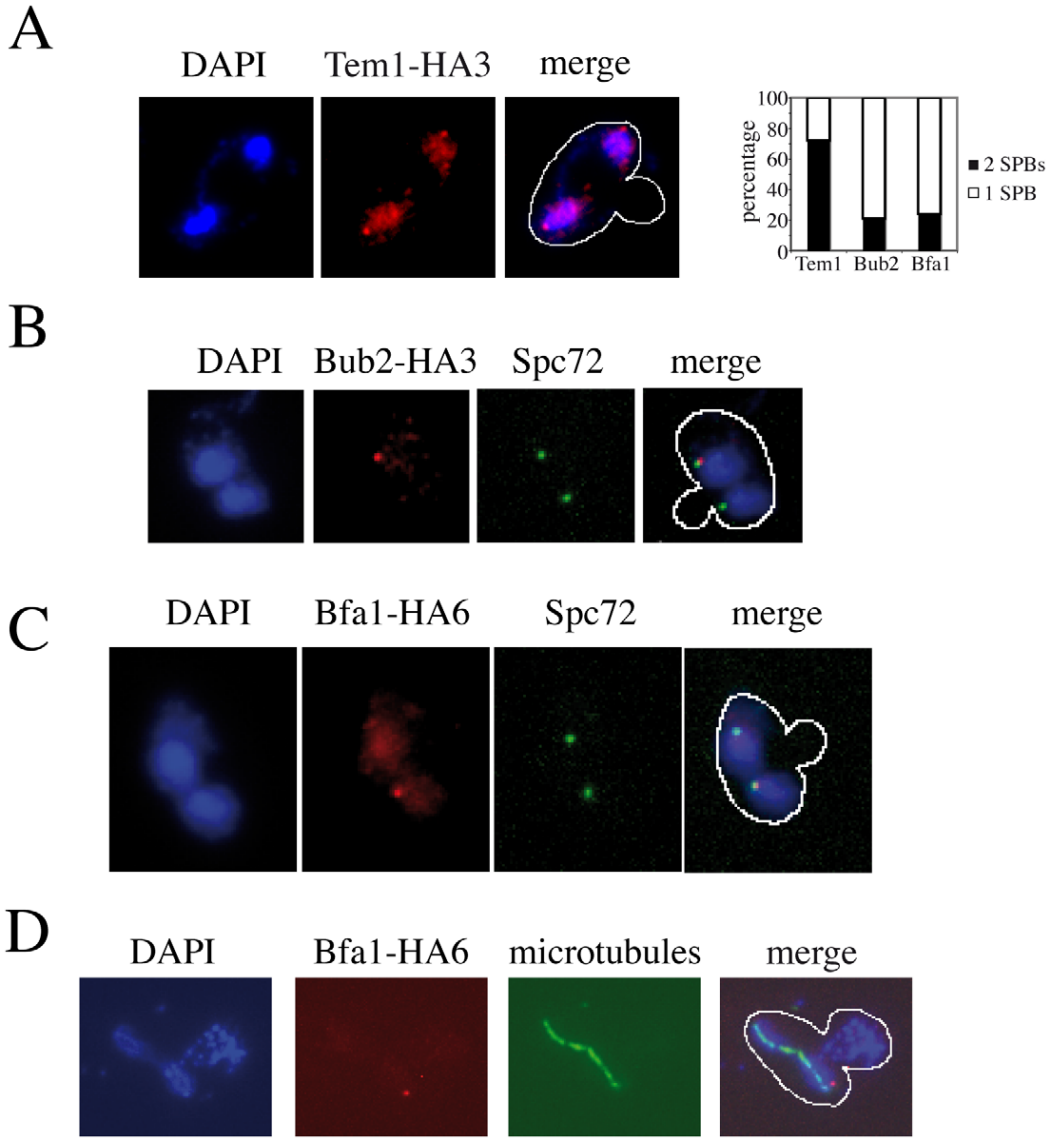
Asymmetric SPB localization of Bub2/Bfa1 in *dma1Δ dma2Δ cla4-75 swe1Δ* cells with mispositioned spindle. A–C: Cells were grown in YEPD at 25°C and shifted to 37°C for 3 hours, followed by nuclear staining with DAPI and visualization of the indicated proteins and of the SPB component Spc72 by indirect immunofluorescence. Only micrographs of cells with mispositioned spindles are shown. A: *dma1Δ dma2Δ cla4-75 swe1Δ* cells (ySP7789) expressing HA-tagged Tem1 (Tem1-HA3). B: *dma1Δ dma2Δ cla4-75 swe1Δ* cells (ySP7771) expressing HA-tagged Bub2 (Bub2-HA3). C: *dma1Δ dma2Δ cla4-75 swe1Δ* cells (ySP7819) expressing HA-tagged Bfa1 (Bfa1-HA6). D: *dma1Δ dma2Δ cla4-75 swe1Δ* cells (ySP7819) expressing Bfa1-HA6 were grown in synthetic medium lacking uracil at 25°C, synchronized in G1 by alpha factor and released at 37°C, followed by nuclear staining with DAPI and visualization of Bfa1-HA6 and microtubules by indirect immunofluorescence. Micrographs were taken 90 minutes after release, when the percentage of mispositioned anaphase spindles was maximal.

We then analysed the subcellular distribution of Bub2, Bfa1 and Tem1, all tagged with HA epitopes at the C-terminus, in *dma1Δ dma2Δ cla4-75 swe1Δ* cells that underwent anaphase in the presence of mispositioned spindles. After 3 hours at the non-permissive temperature, about 30–40% of the cells had undergone anaphase within the mother cell due to spindle misalignment. Tem1-HA3 localized on both SPBs in 72% of these cells (Figure 5A), as expected, whereas Bub2-HA3 and Bfa1-HA6 were retained on both SPBs only in 21% and 24% of cells undergoing anaphase within the mother cell, respectively (Figure 5B, 5C). To exclude that asymmetric SPB localization of Bub2/Bfa1 under these conditions was due to the unscheduled mitotic exit of *dma1Δ dma2Δ cla4-75 swe1Δ* cells, we double stained Bfa1-HA6 and tubulin by immunofluorescence in *dma1Δ dma2Δ cla4-75 swe1Δ* cells released from G1 arrest at 37°C (Figure 5D). Under these conditions, Bfa1-HA6 was present only on one SPB in 77% of the cells undergoing anaphase with mispositioned spindle before spindle disassembly, i.e. before mitotic exit. Thus, Dma proteins and/or Cla4 are required to maintain the Bub2/Bfa1 complex on both SPBs upon spindle mispositioning. However, this might not be the only reason for *dma1Δ dma2Δ cla4-75 swe1Δ* cells to escape mitosis in the presence of mispositioned spindles. In fact, constitutive recruitment of Bub2/Bfa1 to both SPBs by expression of the symmetrically localized Bub2-myc9 variant [37] did not rescue the SPOC defect of these cells (data not shown). Therefore, unscheduled MEN activation in these cells appears to be triggered by means other than mislocalization of Lte1 and Bub2/Bfa1.

### Dma1 and Dma2 control bud neck localization of the Elm1 kinase and phosphorylation of its target Kin4

**Figure 6:**
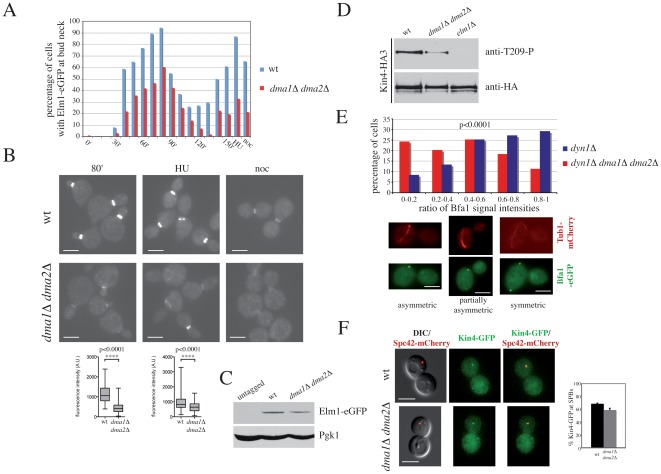
Dma proteins control Elm1 recruitment to the bud neck and Kin4 activation. A, B: Wild type (ySP8813) and *dma1Δ dma2Δ* (ySP8829) cells expressing Elm1-eGFP were arrested in G1 by alpha factor and released into the cell cycle at 25°C (time 0). The culture was split in three parts: one part was left untreated, while the other two were incubated for 150 minutes with HU (120 mM) and nocodazole (15 µg/ml), respectively. Cell samples from the untreated culture were withdrawn at the indicated times for FACS analysis of DNA contents (Figure S6A) and to score budding, nuclear division and bud neck localization of Elm1-eGFP (A and Figure S6B). Representative micrographs were taken for all cultures (B). Fluorescence intensity was quantified in 100 cells for each strain at time 80′ for untreated cells and 150′ for HU-treated cells, and values were plotted in the bottom graphs. Scale bars: 5 µm. C: Western blot with anti-GFP antibodies of cell extracts from untagged cells or wild type (ySP8813) and *dma1Δ dma2Δ* (ySP8829) cells expressing Elm1-eGFP. Pgk1: loading control. D: HA-tagged Kin4 (Kin4-HA3) was immunoprecipitated with anti-HA beads from extracts derived from cycling cultures of wild type (ySP8986), *dma1Δ dma2Δ* (ySP8987) and *elm1Δ* (ySP8997) cells. Immunoprecipitates were subjected to western blot analysis with anti-T209-P [38] and anti-HA antibodies. E: *dyn1Δ* (ySP9493) and *dyn1Δ dma1Δ dma2Δ* (ySP9492) cells expressing Bfa1-eGFP and Tub1-mCherry were grown at 25°C and then shifted to 14°C for 16 hours. Fluorescence intensities of Bfa1-eGFP signals at the two poles of misaligned spindles were quantified on max projected images and plotted as ratios between the less and the most fluorescent signal (ratio = 0: complete asymmetry; ratio = 1: complete symmetry; n>98). Scale bars: 5 µm. F: Wild type (ySP8995) and *dma1Δ dma2Δ* (ySP8994) cells expressing Kin4-GFP and Spc42-mCherry were arrested in mitosis with nocodazole. Z-projected images of representative cells with Kin4-GFP co-localizing with Spc42-mCherry are shown. The histograms on the right side show the quantification of data with standard error bars from three independent repeats. 150–250 cells were scored for each strain in each experiment. Scale bars: 5 µm.

The asymmetric localization of Bub2/Bfa1 on mispositioned spindles was highly reminiscent of that caused by lack of the Kin4 kinase [28]. The Elm1 kinase, besides controlling septin localization [10], [12], [41], [53], has been recently shown to participate in the SPOC by activating Kin4 [38], [39]. We therefore investigated whether Dma proteins regulate Elm1 bud neck localization, which is thought to be important for SPOC signalling [39]. Upon release from G1 arrest, wild type and *dma1Δ dma2Δ* cells progressed similarly through the cell cycle, as shown by their kinetics of DNA replication (Figure S6A), budding and nuclear division (Figure S6B). In sharp contrast, Elm1-eGFP localization at the bud neck was severely impaired in *dma1Δ dma2Δ* cells compared to wild type cells. Indeed, the fraction of cells with Elm1-eGFP at the bud neck was much lower in *dma1Δ dma2Δ* than in wild type cells throughout the experiment (Figure 6A–6B, Figure S6B). In addition, the fluorescence intensity of bud neck-localized Elm1-eGFP was dramatically reduced by lack of Dma proteins (469±288 arbitrary units in *dma1Δ dma2Δ* vs. 1097±426 in wild type at time 80′, *t*-test: p<0,0001; Figure 6B). Similarly, upon release of G1 cells in the presence of hydroxyurea (HU) or nocodazole the bud neck localization of Elm1-eGFP was significantly compromised in the absence of the Dma proteins (Figure 6A and 6B; average fluorescence intensity in HU: 674±335 in *dma1Δ dma2Δ* vs. 928±485 in wild type cells, *t*-test: p<0,0001). The difference in Elm1 recruitment to the bud neck in *dma1Δ dma2Δ* relative to wild type cells was even more dramatic upon longer incubation in HU, where only 2–3% of *dma1Δ dma2Δ* cells display bud neck localization of Elm1-eGFP (data not shown). Similar levels of Elm1-eGFP were found in wt and *dma1Δ dma2Δ* cells (Figure 6C). Furthermore, an a HA-tagged Elm1 variant (Elm1-HA3) was expressed at similar levels in wild type and *dma1Δ dma2Δ* cells throughout the cell cycle (Figure S6C), suggesting that inefficient recruitment of Elm1 to the bud neck in *dma1Δ dma2Δ* cells is not due to decreased protein levels.

To establish if the impaired septin ring stability of *dma* mutant cells was responsible for the inefficient recruitment of Elm1 to the bud neck, we analysed the localization of the Kcc4 kinase, which undergoes cell cycle- and septin-dependent localization at the bud neck [45]. We found that a Kcc4-eGFP fusion was recruited to the bud neck with similar kinetics and efficiency in wild type and *dma1Δ dma2Δ* cells (Figure S7), suggesting that septin defects are unlikely to be responsible for Elm1 mislocalization in the absence of Dma proteins.

### Dma proteins regulate Elm1-dependent activation of the Kin4 kinase

Elm1 activates Kin4 by direct phosphorylation of T209 in the activation loop [38]. We therefore asked whether Kin4 T209 phosphorylation was impaired upon lack of Dma proteins. Strikingly, an antibody specific for T209 phosphorylation [38] recognized lower levels of HA-tagged Kin4 (Kin4-HA3) immunoprecipitated from *dma1Δ dma2Δ* cells compared to wild type cells (Figure 6D). Kin4-T209 phosphorylation, however, was not completely abolished in *dma* mutant cells, while it was undetectable in the absence of Elm1.

*KIN4* overexpression is toxic and delays mitotic exit in otherwise unperturbed conditions due to constitutive activation of Bub2/Bfa1. Deletion of *BUB2* and *BFA1*, as well as of *ELM1*, relieves MEN inhibition and allows cell proliferation in the presence of high levels of Kin4 [25], [38], [39]. Although deletion of *DMA1* and *DMA2* did not rescue the lethality caused by *KIN4* overexpression (Figure S8C), it advanced mitotic exit (as assessed by spindle disassembly) in these conditions (Figure S8B). However, *KIN4*-overexpressing *dma1Δ dma2Δ* cells did not divide efficiently, as shown by the slow reappearance of cells with 1C DNA contents after mitotic exit (Figure S8A), suggesting that high levels of *KIN4* might cause lethality by affecting processes other than mitotic exit.

As Kin4 controls the residence time of Bub2/Bfa1 at the SPBs of misaligned spindles [28], we carefully measured the effect of the absence of Dma proteins on the fluorescence intensity of Bfa1-eGFP signals at spindle poles of mispositioned spindles caused by deletion of the dynein gene *DYN1* [54]. Consistent with a diminished Kin4 activity, Bfa1 distribution at the SPBs of misaligned spindles was significantly more asymmetric in *dyn1Δ dma1Δ dma2Δ* than in *dyn1Δ* cells with functional Dma proteins (Figure 6E; p<0.0001, *t*-test, n>98). Thus, Dma proteins contribute to sustain full Kin4 activation.

The defective Kin4 phosphorylation in the absence of Dma proteins, together with the notion that bud neck localization of Elm1 requires its own kinase activity [53], prompted us to test if Dma proteins affect Elm1 kinase activity. However, *in vitro* autophosphorylation assays on Elm1-HA3 immunoprecipitates showed that the overall Elm1 kinase activity was unaffected upon *DMA1* and *DMA2* deletion (Figure S9A).

Localization of Kin4 at SPBs, which is crucial for its SPOC function [27], is controlled by Rts1 but not by Elm1 [38], [39], [40]. We therefore analysed SPB localization of GFP-tagged Kin4 in wild type and *dma1Δ dma2Δ* cells arrested in mitosis by nocodazole. A similar fraction of wild type and *dma1Δ dma2Δ* cells showed colocalization of Kin4-GFP with the SPB marker Spc42-mCherry (Figure 6F), indicating that Dma1 and Dma2 do not influence Kin4 recruitment to the SPBs in conditions that activate the SPOC. In addition, Dma proteins do not appear to significantly affect Kin4 protein levels (Figure S9B). Thus, Dma proteins are likely implicated in this checkpoint by contributing to full activation of Elm1.

### Defects in spindle positioning and SPOC of *dma1Δ dma2Δ* cells are not suppressed by lack of PP2A^Rts1^

**Figure 7:**
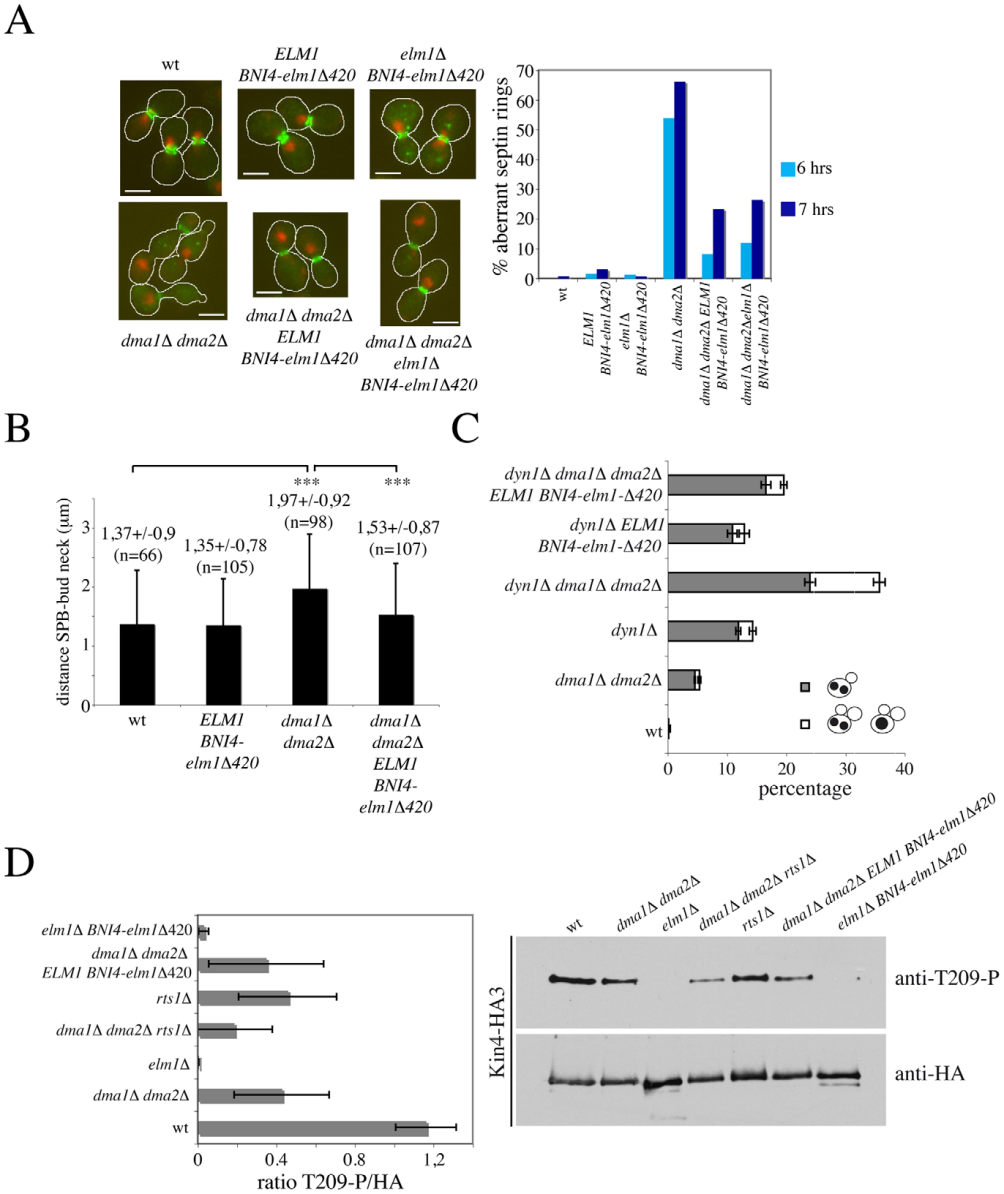
Restoring Elm1 localization at the bud neck of *dma1Δ dma2Δ* cells rescues their septin and SPOC defects without restoring Kin4 phosphorylation. A: Exponentially growing cultures of wild type (W303), *dma1Δ dma2Δ* (ySP1569), *ELM1 BNI4-elm1Δ420* (ySP9589), *elm1Δ BNI4-elm1Δ420* (ySP9599), *dma1Δ dma2Δ ELM1 BNI4-elm1Δ420* (ySP9587) and *dma1Δ dma2Δ elm1Δ BNI4-elm1Δ420* (ySP9588) were treated with HU at 25°C and fixed after 6 and 7 hours to analyse the septin ring by in situ immunofluorescence with anti-Cdc11 antibodies. Representative micrographs were taken at t = 7 hrs. Scale bars: 5 µm. B: Average SPB distance from the bud neck was measured in logarithmically growing wild type (ySP9594), *dma1Δ dma2Δ* (ySP9593), *ELM1 BNI4-elm1Δ420* (ySP9636) and *dma1Δ dma2Δ ELM1 BNI4-elm1Δ420* (ySP9634) strains expressing Spc42-mCherry to visualize the SPB. Only budded cells with two SPBs facing eachother in the mother cell were scored as indicative of a pre-anaphase stage and distances were measured from the most proximal SPB to the bud neck. ***: p<0,001, *t*-test. C: wild type (W303), *dma1Δ dma2Δ* (ySP1569), *dyn1Δ* (ySP6292), *dyn1Δ dma1Δ dma2Δ* (ySP7454), *dyn1Δ ELM1 BNI4-elm1Δ420* (ySP9590), and *dyn1Δ dma1Δ dma2Δ ELM1 BNI4-elm1Δ420* (ySP9592) cells were shifted to 14°C for 16 hours, followed by scoring cell morphology and nuclear division after nuclear staining with propidium iodide. Histograms represent average values of three independent experiments, with bars showing standard errors. At least 500 cells were scored in each experiment. D: HA-tagged Kin4 (Kin4-HA3) was immunoprecipitated with anti-HA beads from extracts derived from cycling cultures of wild type (ySP8986), *dma1Δ dma2Δ* (ySP8987), *elm1Δ* (ySP8997), *dma1Δ dma2Δ rts1Δ* (ySP9484), *rts1Δ* (ySP9485), *dma1Δ dma2Δ ELM1 BNI4-elm1Δ420* (ySP9487) and *elm1Δ BNI4-elm1Δ420* (ySP9489) cells. Immunoprecipitates were subjected to western blot analysis with anti-T209-P [38] and anti-HA antibodies. The graph represents mean ratios with standard error bars between phosphorylated and total Kin4-HA3 in the immunoprecipitates of three independent experiments.

The antagonistic function of Dma proteins and PP2A^Rts1^ on septin ring stability prompted us to test if this antagonism also applies to other processes implicating these proteins, such as spindle positioning and SPOC. First we asked if *RTS1* deletion could rescue the spindle positioning defects of *dma1Δ dma2Δ* cells. To this end we measured the mean distance between the bud neck and the most proximal SPB in budded cells with short bipolar spindles (Figure S3C). Spindle alignment was impaired in *dma1Δ dma2Δ* cells independently of the presence of *RTS1*, indicating that PP2A^Rts1^ does not antagonize Dma proteins in spindle positioning. We then asked if lack of Dma proteins and PP2A^Rts1^ might compensate each other in SPOC signalling. When we tried to combine *DMA1* and *DMA2* deletion with that of *RTS1* in cells lacking the dynein gene *DYN1* to increase spindle misalignment, we found that the quadruple *dyn1Δ dma1Δ dma2Δ rts1Δ* mutant was unviable. We therefore analysed SPOC defects in cells deleted for *KAR9* that also undergo frequent spindle misalignment [55]. *DMA1/2* and *RTS1* deletion did not suppress each other's SPOC failure (Figure S3D), suggesting that PP2A^Rts1^ does not counteract the function of Dma proteins in the SPOC. Consistently, analysis of Kin4 phosphorylation on T209 showed that *RTS1* deletion impaired Kin4-T209 phosphorylation in an additive manner with deletion of *DMA1* and *DMA2* (Figure 7D). In addition, the defective localization of Kin4 at SPBs of misaligned spindles in *rts1Δ* cells was not suppressed by lack of Dma proteins (Figure S3E). Therefore, PP2A^Rts1^ and Dma proteins do not have antagonistic functions in spindle positioning and SPOC.

### Septin and SPOC defects of *dma1Δ dma2Δ* cells are rescued by artificial targeting of Elm1 to the bud neck

The ability of *dma1Δ dma2Δ cla4-75 swe1Δ* cells to exit mitosis in spite of spindle mispositioning (Figure 4) raised the possibility that Swe1 could be involved in the SPOC. We analysed SPOC proficiency in *dyn1Δ* cells at low temperature to increase the fraction of cells undergoing anaphase and nuclear division within the mother cell due to spindle misalignment [54]. Nuclear division in the mother cell was scored as indicative of spindle position defects, while re-budding of these cells indicated the failure to activate the SPOC. Under these conditions, 15–25% of *dyn1Δ* cells underwent nuclear division in the mother cell, but only a minor fraction of them re-budded (Figure S5A, S5C, S5D), indicating that the SPOC efficiently prevented mitotic exit. In agreement with our previous data [42], deletion of *DMA1* and *DMA2* enhanced the spindle position defect caused by loss of Dyn1 and allowed a fraction of cells to exit mitosis and re-bud in the presence of mispositioned spindles. *SWE1* deletion did not overcome the mitotic exit delay of *dyn1Δ* cells and did not enhance the SPOC defect of *dyn1Δ dma1Δ dma2Δ* cells (Figure S5A), suggesting that Swe1 does not influence the SPOC response. With a similar experiment we showed that *LTE1* deletion did not suppress the SPOC defect of *dyn1Δ dma1Δ dma2Δ* cells (Figure S5C), confirming that unscheduled mitotic exit upon spindle misalignment in the absence of Dma proteins does not require Lte1. Consistently, Lte1 was localized at the bud cortex with wild type kinetics during the cell cycle in *dma1Δ dma2Δ* cells (Figure S5B).

If the major function of Dma proteins in septin ring stabilization, spindle positioning and the SPOC were to promote efficient localization of Elm1 at the bud neck, artificial recruitment of Elm1 to the bud neck should suppress all defects caused by *DMA1* and *DMA2* deletion. To test this idea, we then took advantage of a Bni4-Elm1Δ420 chimeric protein, where a truncated Elm1 kinase unable to localize at the bud neck was fused to the Bni4 bud neck protein [39]. In agreement with published data [39], we could confirm that Bni4-Elm1Δ420 suppressed the SPOC defect of *dyn1Δ elm1Δ* cells (data not shown). Strikingly, this chimeric protein was able to compensate for the lack of Dma proteins in all above processes. First, it suppressed the lethality of *dma1Δ dma2Δ elm1Δ* cells (data not shown) and septin mislocalization in HU-treated *dma1Δ dma2Δ* cells (Figure 7A). Second, it restored efficient spindle alignment in *dma1Δ dma2Δ* cells (Figure 7B). Third, it rescued the SPOC defects of *dyn1Δ dma1Δ dma2Δ* cells (Figure 7C). Interestingly, though, we found that Bni4-Elm1Δ420 was dramatically deficient at phosphorylating Kin4 on T209 and could not increase Kin4-T209 phosphorylation in *dma1Δ dma2Δ* cells (Figure 7D). Thus, Bni4-Elm1Δ420 might re-establish a normal SPOC response in the absence of Dma proteins by mechanism(s) other than restoring full Kin4 activity. Consistently, expression of the phosphomimetic mutant variant Kin4-T209D [39], did not rescue the SPOC defect of *dyn1Δ dma1Δ dma2Δ* cells (Figure S5D).

Altogether, these data indicate that Elm1 bud neck localization is a key determinant in SPOC integrity and Dma proteins finely tune this process.

## Discussion

### Role of the Dma proteins in septin ring dynamics

The septin ring oscillates between a frozen state, which lasts for most cell cycle, and a fluid state, which is highly dynamic and is observed in late G1 and during septin ring splitting at cytokinesis onset (reviewed in [4]). The transition from fluid to frozen state is promoted by the Cla4 and Gin4 kinases, which directly phosphorylate septins [8], [11], while PP2A^Rts1^ is required for the opposite transition [8]. We previously implicated Dma1 and Dma2 in the regulation of septin ring organization [42], and we now extended our initial observations by showing through different approaches that indeed Dma proteins promote septin ring stabilization. In fact, *DMA1* and *DMA2* double deletion destabilizes the septin ring, as shown by FRAP, and is synthetically lethal with mutations that destabilize the septin ring. Conversely, mutations destabilizing the septin ring, such as *cdc12-1* or *SHS1* deletion, can alleviate the toxic effects of *DMA2* overexpression, which causes the formation of a new septin ring at the incipient bud site before the old one has split. Moreover, *dma1 dma2 cla4* triple mutants are unviable and unable to deposit and maintain the septin ring at the bud neck, but the lack of PP2A^Rts1^ is sufficient to rescue their lethality and to restore an apparently normal septin ring at the bud neck. We find that *RTS1* deletion can suppress the septin defects caused by lack of Cla4 and, to a lesser extent, of Dma1/2, which is totally consistent with the notion that PP2A^Rts1^ inactivation stabilizes the septin ring [8]. Although *RTS1* deletion partially suppresses septin ring dynamics in *dma1Δ dma2Δ* mutants, it does not rescue their spindle positioning defects. As an intact septin ring is required for proper spindle positioning [16], it is possible that the septin ring is not fully functional in *dma1Δ dma2Δ rts1Δ* mutants in spite of its apparently normal look. Consistently, *dma1Δ dma2Δ rts1Δ* cells grow more slowly and have a reduced maximal permissive temperature than *dma1Δ dma2Δ* or *rts1Δ* cells.

How do Dma proteins control septin dynamics? Dma1 and Dma2 are E3 ubiquitin ligases that can form in vitro both K48- and K64-linked polyubiquitin chains [56], suggesting that they might be involved in both proteolytic and regulatory ubiquitylation. Therefore, Dma proteins might directly ubiquitylate septins or their regulators to control septin turnover within the ring. However, septins could not be found ubiquitylated in a previous study [57] and our efforts in this direction did not provide reproducible results (data not shown). Moreover, the septin ring instability of *dma1Δ dma2Δ* mutants does not appear to be caused by increased Rts1 protein levels. Our finding that Elm1 localization at the bud neck is defective in cells lacking Dma proteins suggests a possible mechanism for regulation of septin dynamics by these proteins. Indeed, similarly to Dma1 and Dma2, Elm1 is required for cytokinesis and proper localization of septins at the bud neck [10], [12], [41], [53]. In addition, its inactivation, like that of Dma proteins [46], causes a Swe1-dependent mitotic delay [12]. The impaired Elm1 localization in *dma* mutant cells is not a mere consequence of their defects in septin dynamics. In fact, recruitment of the Kcc4 and Hsl1 kinases to the bud neck is unaffected upon *DMA1/2* deletion (this manuscript and [46]), whereas Swe1 [46] and the polo kinase Cdc5 (data not shown) are present at higher, rather than lower, levels at the bud neck of *dma1Δ dma2Δ* cells relative to wild type. At a first glance, the synthetic lethality between *ELM1* and *DMA1/DMA2* deletion seems at odds with the proposal that Elm1 and Dma proteins might act in the same pathway. However, Elm1 was found to regulate Cla4, Gin4 and Hsl1 [12], [13], [58], which in turn become essential in the absence of Dma1 and Dma2. Furthermore, *ELM1* deletion also causes synthetic growth defects when combined with *HSL1*, *CLA4* or *GIN4* deletion [10], [12], making functional relationships difficult to assess on the basis of genetic interactions. Therefore, Dma proteins might regulate a subset of Elm1 functions that are independent of Gin4 and Hsl1 regulation. Nonetheless, Elm1 regulation by Dma proteins is likely to take place at the bud neck. In fact, artificial recruitment of Elm1 to the bud neck through the chimeric Bni4-Elm1Δ420 protein is sufficient to rescue the lethality of *dma1Δ dma2Δ elm1Δ* cells and to efficiently suppress the formation of aberrant septin rings in HU-treated *dma1Δ dma2Δ* cells. In addition, Bni4-Elm1D420 also restores proper spindle positioning in *dma1Δ dma2Δ* cells, strongly suggesting that septin defects and spindle mispositioning in these mutants stem from the same primary defect. However, Bni4-Elm1Δ420 does not suppress the lethality of *dma1Δ dma2Δ cla4Δ* mutants (data not shown), raising the possibility that Dma proteins target additional proteins, besides Elm1, for septin regulation.

How do Dma proteins regulate Elm1 recruitment to the bud neck? One possibility is that they do so through direct Elm1 ubiquitylation. Although at the moment we cannot completely rule out this hypothesis, we found that Elm1 is ubiquitylated in vivo, but its overall ubiquitylation does not seem to require Dma proteins (data not shown). Therefore, the identification of the critical ubiquitylation target of Dma1/2 for efficient Elm1 recruitment to the bud neck will be a challenging task for the future.

### Dma-dependent regulation of the SPOC

The failure of *dma1Δ dma2Δ cla4-75 swe1Δ* cells to activate the SPOC in the presence of mispositioned spindles is likely to be ascribed to the sole lack of Dma1 and Dma2 for three main reasons: i) lack of Dma proteins is sufficient to cause re-budding of *dyn1Δ* and *kar9Δ* cells that undergo spindle mispositioning (this manuscript and [42]); ii) Cla4 promotes, rather than inhibits, timely mitotic exit and MEN activation independently of Swe1 [50], [51], [52]; iii) Swe1 does not appear to be involved in the SPOC.

The SPOC defect of *dma* mutant cells could in principle arise from their septin defects. Indeed, septin mutants were shown to be defective in the SPOC presumably as a consequence of Lte1 spreading into the cytoplasm, since their ability to escape mitosis in the presence of mispositioned spindles depends on Lte1 [36]. In contrast, *dma1Δ dma2Δ* cells localize properly Lte1 at the bud cortex and exit mitosis in the presence of misaligned spindles independently of Lte1, suggesting that Dma proteins control mitotic exit through a distinct mechanism.

Our finding that PP2A^Rts1^ counteracts the function of Dma proteins in regulating septin dynamics raised the possibility that these proteins antagonize each other in SPOC signalling. Although its exact role in the SPOC remains to be established, PP2A^Rts1^ is required for Kin4 localization at the SPB and cell cortex [38], [40]. Recent data indicating that *LTE1* deletion is sufficient to restore proper Kin4 localization and SPOC response in *rts1Δ* cells raise the possibility that PP2A^Rts1^ might control Lte1 [33]. PP2A^Rts1^ was shown to reverse Elm1-dependent Kin4 hyperphosphorylation [38], [40]. In addition, *RTS1* deletion rescues the hyperpolarized growth of *elm1Δ* cells [47], suggesting that PP2A^Rts1^ counteracts some Elm1 functions. Surprisingly, however, we find that Elm1-dependent Kin4 phosphorylation on T209 is somewhat reduced in the absence of *RTS1* compared to wild type levels, suggesting that the role of PP2A^Rts1^ in the SPOC is likely very complex. Our data indicate that PP2A^Rts1^ does not antagonize Dma proteins' function in the SPOC and viceversa. In fact, the levels of Kin4-T209 phosphorylation are lower in *dma1Δ dma2Δ rts1Δ* cells than they are in *dma1Δ dma2Δ* or *rts1Δ* mutants. Furthermore, deletion of *RTS1* does not rescue the SPOC defect of *kar9Δ dma1Δ dma2Δ* cells and the quadruple *dyn1Δ dma1Δ dma2Δ rts1Δ* mutant is unviable, whereas *dyn1Δ dma1Δ dma2Δ* and *dyn1Δ rts1Δ* mutants are viable.

Our data indicate that Elm1 mislocalization is the main, if not only, reason for the SPOC failure of *dma* mutant cells. In fact, artificial recruitment of Elm1 to the bud neck through the Bni4-Elm1Δ420 chimera is sufficient to re-establish a proper checkpoint response in *dyn1Δ dma1Δ dma2Δ* cells, as it does in *dyn1Δ elm1Δ* cells [39]. These results are also in agreement with the proposal that bud-neck localized Elm1 is important for SPOC signalling [39]. Although Elm1 has an established role in the SPOC through Kin4 phosphorylation 38,39, we propose that the fraction of Elm1 at the bud neck participates to the SPOC by phosphorylating targets other than Kin4. Indeed, we find that the Bni4-Elm1Δ420 chimeric protein is almost completely defective at phosphorylating Kin4 on T209 in the activation loop, suggesting that the suppression of SPOC defects of *dyn1Δ dma1Δ dma2Δ* and *dyn1Δ elm1Δ* cells is due to a mechanism distinct from Kin4 phosphorylation. This conclusion is in agreement with the earlier observation that bud neck-localized Elm1 is not required for Kin4 phosphorylation and activation [38], although the C-terminus of Elm1, which mediates its bud neck recruitment, is necessary for SPOC response [39]. The finding that a phosphomimetic Kin4-T209D variant does not rescue the SPOC defects of *dma1Δ dma2Δ* cells is also entirely consistent with the above conclusion.

Why *dma1Δ dma2Δ* mutants are defective in Kin4 phosphorylation on T209 remains to be established. It is possible that Elm1 carries at its C terminus important determinants for both its bud neck localization and Kin4 phosphorylation that are misregulated in the absence of Dma proteins. In any case, Bni4-Elm1Δ420 is sufficient to proficiently support SPOC signalling without proper levels of Kin4 phosphorylation, which is normally strictly required for SPOC response [38], thus raising the possibility that this chimeric protein might have some dominant property over Kin4 in the regulation of mitotic exit.

The low levels of Elm1-dependent Kin4 phosphorylation in *dma1Δ dma2Δ* cells likely result in reduced levels of Kin4 kinase activity, which requires T209 phosphorylation by Elm1 [38]. Accordingly, deletion of *DMA1* and *DMA2* suppresses the mitotic exit defects caused by *KIN4* overexpression. In addition, as Kin4 is required in the mother cell to maintain symmetric localization of Bub2/Bfa1 on misaligned spindles [28], low Kin4 kinase levels in cells lacking Dma proteins would explain the asymmetry of Bub2/Bfa1 at the spindle poles of mispositioned spindles in these cells.

**Figure 8:**
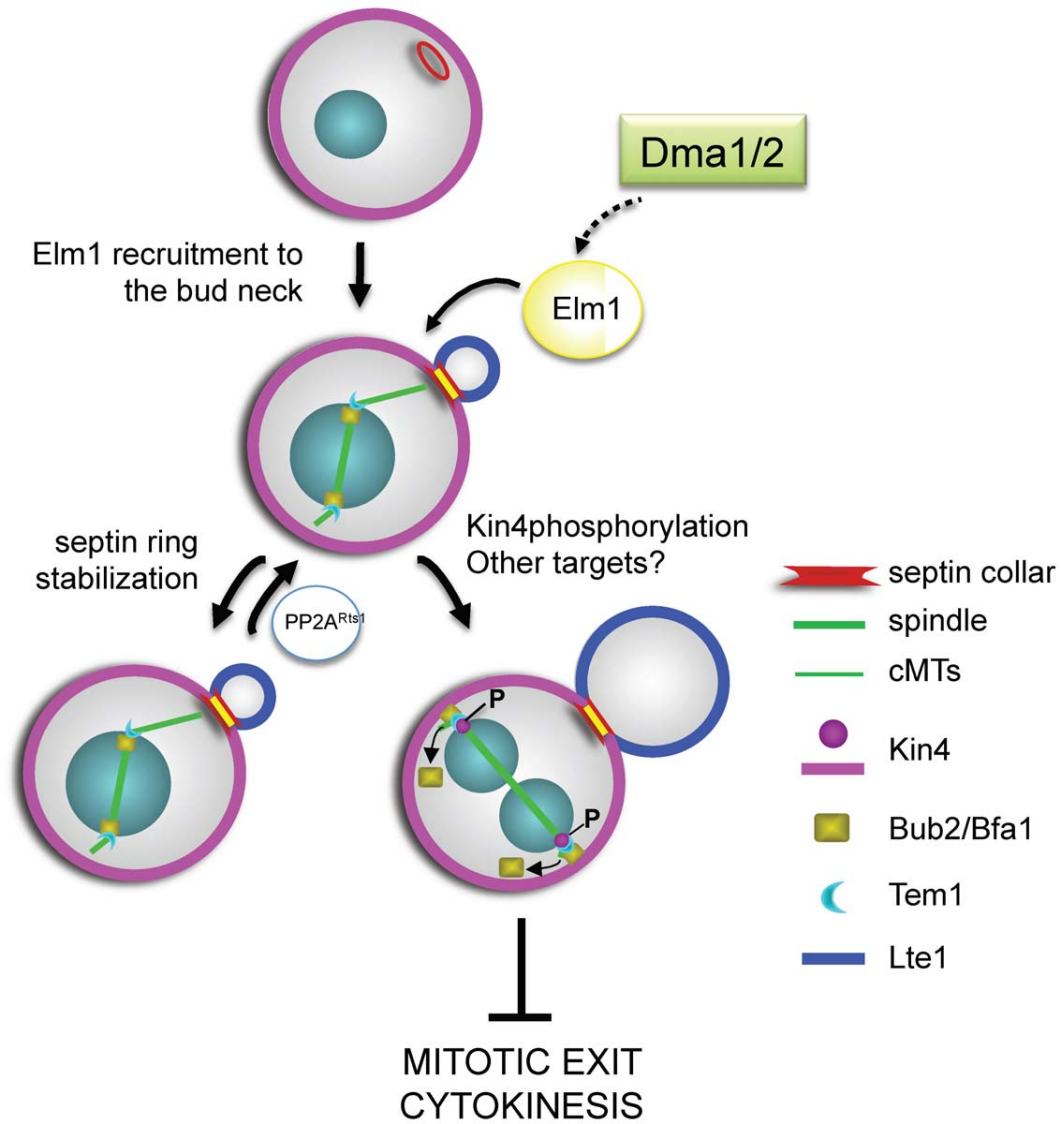
A model for the role of Dma proteins in regulation of septin dynamics and SPOC response. Dma1 and Dma2 control Elm1 recruitment to the bud neck and this promotes both septin ring stability and inhibition of mitotic exit upon spindle mispositioning through phosphorylation and activation of Kin4 and probably of additional targets. PP2A^Rts1^ counteracts Elm1 functions in septin ring stabilization. See text for further details.

Our results together with published data indicate that Dma1/2, Elm1 and Kin4 are part of the same SPOC regulatory module, with Dma proteins controlling Elm1 localization that is in turn required for full Kin4 activation (see below, Figure 8). It is worth noting that Dma1/2, Kin4 and Elm1 share an important feature, as they all are required to prevent mitotic exit in response to spindle mispositioning but not to spindle depolymerization, unlike Bub2 and Bfa1 [25], [39], [42]. Our data also strongly suggest a tight link between disrupted integrity of septin ring dynamics and SPOC defects in *dma1Δ dma2Δ* cells, which is consistent with the proposal that the septin ring at the bud neck serves as a platform for the signalling mechanisms that inhibit mitotic exit in response to spindle mispositioning [36].

Based on the above observations, we propose a model for SPOC signalling depicted in Figure 8, where Dma1 and Dma2 regulate both septin ring dynamics and the SPOC by promoting the efficient recruitment of Elm1 to the bud neck. Elm1 activity on one side stabilizes the septin ring, with PP2A^Rts1^ counteracting this process, and on the other leads to activation of Kin4 through phosphorylation on T209. Kin4 in turn regulates the turnover of Bub2/Bfa1 [28] and prevents Bfa1 inhibitory phosphorylation by the polo kinase [27], thus maintaining the GAP active towards Tem1 and inhibiting mitotic exit and cytokinesis.

Interestingly, the human Dma homolog Rnf8 localizes at the spindle midbody and has been proposed to antagonize the human mitotic exit network [59], suggesting that it might be the functional human counterpart of Dma proteins. The identification of the ubiquitylation targets of Dma proteins will be a challenging task for future research and will help to elucidate the mechanism(s) through which they control cytokinesis and help maintaining genome stability.

## Materials and Methods

### Strains, media and reagents, genetic manipulations

All yeast strains (Table S1) were derivatives of or were backcrossed at least six times to W303 (*ade2-1, trp1-1, leu2-3,112, his3-11,15, ura3, ssd1*). Cells were grown in either synthetic minimal medium supplemented with the appropriate nutrients or YEP (1% yeast extract, 2% bactopeptone, 50 mg/l adenine) medium supplemented with 2% glucose (YEPD), 2% raffinose (YEPR) or 2% raffinose and 2% galactose (YEPRG). Unless differently stated, alpha factor was used at 2 µg/ml, HU at 150 mM and nocodazole at 15 µg/ml. For galactose induction of synchronized cells 1% galactose was added to YEPR half an hour before release from alpha factor.

Standard techniques were used for genetic manipulations [60], [61]. Gene deletions were generated by one-step gene replacement [62]. One-step tagging techniques [63], [64] were used to create -HA3 tagged Elm1, Rts1 and Kin4, as well as and -eGFP tagged Elm1, Kcc4 and Bfa1.

### FRAP and fluorescence microscopy

FRAP experiments were performed as previously described [8] on a Zeiss LSM 510 confocal. Essentially, logarythmically growing cells expressing GFP-Cdc12 were grown overnight in YEPD, resuspended in synthetic complete medium and spread on 2% agar pads. Half the septin ring was bleached with a sequence of 20 to 25 irradiations at 50% of laser intensity. Fluorescence intensities were analyzed with ImageJ. Background staining in each cell was subtracted. To correct for general bleaching, fluorescence intensities of septin rings were normalized to those of 2–3 reference cells present in each movie.

For time lapse video microscopy of GFP-Cdc12 cells were imaged at 30°C on agar pellets of complete synthetic medium containing galactose. Movies were recorded with a DeltaVision microscope and a 100× oil immersion objective using the Softworx software (Applied Precision). Individual Z stacks contained 10 planes with a step size of 0.6 mm. Pixels were binned symmetrically by 2. After image acquisition the movies were projected as max intensity projections.

Nuclear division and SPOC proficiency were scored with a fluorescence microscope on cells stained with propidium iodide or DAPI.

In situ immunofluorescence was performed on formaldehyde-fixed cells. Visualization of septin rings was performed using anti-Cdc11 polyclonal antibodies (1∶200, sc-7170 Santa Cruz) followed by indirect immunofluorescence with Alexa Fluor 488-conjugated anti-rabbit antibody (1∶100, Invitrogen). In situ immunofluorescence of HA-tagged Bub2, Bfa1 and Tem1 was carried out as previously described [37]. To detect spindle formation and elongation, anti-tubulin immunostaining was performed with the YOL34 monoclonal antibody (Serotec) followed by indirect immunofluorescence using rhodamine-conjugated anti-rat Ab (1∶100 Pierce Chemical Co). Detection of Spc42-mCherry, Cdc3-GFP, Lte1-GFP, Elm1-eGFP and Kcc4-eGFP was carried out on ethanol-fixed cells, upon wash with PBS and sonication. Simultaneous detection of Bfa1-eGFP and Tub1-mCherry was carried out on cells fixed overnight at −20°C with 3.7% formaldehyde in 0.1 M potassium phosphate buffer pH 6.4. Stacks of 12–20 images (step size 0.3 µm) were taken on unfixed cells washed with PBS to detect co-localization of Kin4-GFP with Spc42-mCherry and of Bfa1-eGFP with Tub1-mCherry. Digital images were taken with an oil 100× 1.3-0,5PlanFluor oil objective (Nikon) with a Leica DC350F charge-coupled device camera mounted on a Nikon Eclipse 600 and controlled by the Leica FW4000 software (Bub2-HA3, Bfa1-HA6, Tem1-HA3, Lte1-GFP, Cdc11) or with a 63× 1,4-0,6 HCX PlanApo objective (Leica) with a Coolsnap HQ2 1 charge-coupled device camera (Photometrics) mounted on a Leica DM6000 fluorescence microscope and controlled by the MetaMorph imaging system software (Elm1-eGFP, Kcc4-eGFP, Kin4-GFP, Bfa1-eGFP). Fluorescence intensity of Elm1-eGFP and Kcc4-eGFP at the bud neck was measured on at least 100 cells for each strain with ImageJ on a single plane that had the bud neck in focus. Fluorescence intensity of Bfa1-eGFP was measured on Z-stacks max projections. Significance of the differences between fluorescence intensities was statistically tested by means of a two-tailed *t*-test, assuming unequal variances.

### Protein extracts, immunoprecipitations, and kinase assays

For immunoprecipitations of Kin4-HA3 and Elm1-HA3, pellets from 50 ml yeast cultures (10^7^ cells/ml) were lysed with acid-washed glass beads in lysis buffer (50 mM Tris-Cl pH 7.5, NaCl 150 mM, 10% glycerol, 1 mM EDTA, 1% NP40, supplemented with protein inhibitors (Complete, Roche) and phosphatase inhibitors (PhoSTOP, Roche). Total extracts were cleared by spinning at 14000 rpm for 10 minutes and quantified by NanoDrop. Same amounts of protein extracts were subjected to immunoprecipitation with an anti-HA affinity matrix (Roche) for Kin4-HA3 or with anti-HA (12CA5) antibodies followed by binding to protein A-sepharose for Elm1-HA3.

For kinase assays, 2.6 mg of total extracts were used to immunoprecipitate Elm1-HA3. Kinase assays were performed essentially as described [65] in 30 µl of kinase buffer (50 mM Tris-Cl pH 7.5, 10 mM MgCl2, 1 mM MnCl2, 1 mM DTT, 10 µCi gP^33^ATP, 10 µM ATP) for 30′ at 30°C.

For Western blot analysis, protein extracts were prepared according to [37]. Proteins transferred to Protran membranes (Schleicher and Schuell) were probed with monoclonal anti-HA, anti-GFP and Pgk1 antibodies (Molecular Probes) or polyclonal Cdc5 antibodies (sc-6733 Santa Cruz). Secondary antibodies were purchased from Amersham and proteins were detected by an enhanced chemiluminescence system according to the manufacturer.

### Other techniques

Flow cytometric DNA quantification was performed according to [66] on a Becton-Dickinson FACScan.

## Supporting Information

Figure S1 Septin ring maintenance throughout the cell cycle requires Cla4 and Dma proteins. A–B: Small unbudded cells of wild type (W303) and *dma1Δ dma2Δ cla4-75* (ySP5247) strains were isolated by centrifugal elutriation and resuspended in fresh medium at the permissive temperature (25°C) at time 0 (upper graphs). Aliquots of these cultures were then shifted to 37°C after 120, 130 and 140 minutes (bottom panels). At the indicated times, cell samples were taken for FACS analysis of DNA contents (not shown), for scoring budding and nuclear division (shown only at 25°C) and for the septin ring analysis as in Figure 1B. Micrographs of representative cells are shown in (B). Scale bars: 5 µm. C: Logarithmically growing cultures of wild type (W303), *dma1Δ dma2Δ cla4-75* (ySP5264) and *cdc12-6* (ySP5182) cells were arrested in mitosis by 2.5 hours nocodazole treatment at 25°C and then shifted to 37°C, followed by FACS analysis of DNA contents (not shown) and in situ immunofluorescence analysis of the septin ring with anti-Cdc11 antibodies at the indicated times. We confirmed that the mitotic arrest was maintained throughout the time course in all cell cultures.
(TIF)

Figure S2 The FHA and RING domains are required for Dma proteins' function in septin ring organization. A: Serial dilutions of stationary phase cultures of the strains with the indicated genotypes were grown in synthetic raffinose medium lacking leucine at 25°C, spotted on YEPRG and incubated at the indicated temperatures for 2 days. dma1*FHA1: dma1-S220A, H223L; dma1*FHA2: dma1-G192E; dma2*RING: dma2-C451S, H456A [67]. B: Strains with the indicated genotypes were grown in synthetic medium lacking leucine at 25°C and arrested in G1 by alpha factor. 1% galactose was added 30 minutes before the release in YEPRG at 37°C. The septin ring was stained by *in situ* immunofluorescence with anti-Cdc11 antibodies and micrographs were taken after 3 hours at 37°C. An asterisk indicates the missense mutations in the Dma RING and FHA domains that are detailed in (A). Scale bars: 5 µm.
(TIF)

Figure S3 Lack of Rts1 does not suppress the spindle positioning and SPOC defects of *dma1Δ dma2Δ* mutants. A: Cycling cultures of wild type (W303), *dma1Δ dma2Δ* (ySP1569), *rts1Δ* (ySP9616) and *dma1Δ dma2Δ rts1Δ* (ySP9617) cells were treated with HU at 25°C and fixed after 6 hours to analyse the septin ring by in situ immunofluorescence with anti-Cdc11 antibodies. Scale bars: 5 µm. B: Serial dilutions of the strains in (A) were spotted on YEPD plates and incubated for 2 days at the indicated temperatures. C: Average SPB distance from the bud neck was measured as in Figure 7B in logarithmically growing cultures of wild type (ySP9594), *dma1Δ dma2Δ* (ySP9593), *rts1Δ* (ySP9596) and *dma1Δ dma2Δ rts1Δ* (ySP9595) cells expressing Spc42-mCherry to visualize SPBs. D: Cycling cultures of wild type (W303), *dma1Δ dma2Δ* (ySP1569), *kar9Δ* (ySP6270), *rts1Δ* (ySP9616), *kar9Δ rts1Δ* (ySP9664), *dma1Δ dma2Δ rts1Δ* (ySP9662), *kar9Δ dma1Δ dma2Δ* (ySP9661) and *kar9Δ dma1Δ dma2Δ rts1Δ* (ySP9663) were shifted to 37°C for 3 hours, followed by scoring cell morphology and nuclear division after nuclear staining with propidium iodide. Histograms represent average values of three independent experiments, with bars showing standard errors. At least 500 cells were scored in each experiment. E: Wild type (ySP8996), *rts1Δ* (ySP9467) *dma1Δ dma2Δ* (ySP8994) and *dma1Δ dma2Δ rts1Δ* (ySP9466) cells expressing Kin4-GFP and Spc42-mCherry were arrested in mitosis with nocodazole. Z-projected images of representative cells with Kin4-GFP co-localizing with Spc42-mCherry are shown. The histograms on the right side show the quantification of data with standard error bars from three independent repeats. At least 100 cells were scored for each strain in each experiment. Note that in *rts1Δ* strains Kin4-GFP signals at SPBs, when present, are always very faint. Scale bars: 5 µm.
(TIF)

Figure S4 *DMA2* overexpression delays septin ring splitting and disassembly. Wild type (upper rows) and *GAL1-DMA2* cells (lower rows) expressing Cdc12-GFP were recorded every 10 minutes by time lapse video microscopy. The arrowhead indicates septin ring splitting; the arrow indicates appearance of a new septin ring. Scale bars: 5 µm.
(TIF)

Figure S5 Lack of Swe1 or Lte1 does not suppress the SPOC defect of *dma1Δ dma2Δ* mutants. A: Exponentially growing cultures of wild type (W303), *dma1Δ dma2Δ* (ySP1569), *swe1Δ* (ySP1370), *dyn1Δ* (ySP6292), *dyn1Δ swe1Δ* (yRF1301), *dyn1Δ dma1Δ dma2Δ* (ySP7454) and *dyn1Δ dma1Δ dma2Δ swe1Δ* (yRF1306) cells were shifted to 14°C for 16 hours, followed by scoring cell morphology and nuclear division after nuclear staining with propidium iodide. Histograms represent average values of three independent experiments, with bars showing standard errors. At least 500 cells were scored in each experiment. B: Cycling cultures of wild type (ySP3333) and *dma1Δ dma2Δ* (ySP4380) strains carrying a *LTE1-GFP* construct under the control of the galactose-inducible *GAL1* promoter were grown in YEPR, arrested in G1 with a factor, pre-induced with 1% galactose for 30 minutes and finally released in YEPRG at 25°C (time = 0). Cells were collected at the indicated time points for FACS analysis of DNA contents (not shown) and localization of Lte1-GFP at the bud cortex (graph). At least 200 cells were scored at each time point. C: Exponentially growing cultures of wild type (W303), *dma1Δ dma2Δ* (ySP1569), *lte1Δ* (ySP8657), *dyn1Δ lte1Δ* (yRF1434), *dyn1Δ* (ySP6292), *dyn1Δ dma1Δ dma2Δ* (ySP7454) and *dyn1Δ dma1Δ dma2Δ lte1Δ* (ySP9523) cells were treated and scored as in (A). D: Cycling cultures of wild type (W303), *dyn1Δ* (ySP6292), *dyn1Δ KIN4-13myc* (ySP9238), *dyn1Δ KIN4-T209D-13myc* (ySP9239), *dma1Δ dma2Δ* (ySP1569), *dyn1Δ dma1Δ dma2Δ* (ySP7454), *dyn1Δ dma1Δ dma2Δ KIN4-13myc* (ySP9236), *dyn1Δ dma1Δ dma2Δ KIN4-T209D-13 myc* (ySP9240), *elm1Δ HSL1-T273E* (ySP9214), *dyn1Δ elm1Δ HSL1-T273E* (ySP9244), *dyn1Δ elm1Δ HSL1-T273E KIN4-13myc* (ySP9289) and *dyn1Δ elm1Δ HSL1-T273E KIN4-T209D-13myc* (ySP9243) cells were treated and scored as in (A).
(TIF)

Figure S6 Elm1 localization and protein levels during the cell cycle in wild type and *dma1Δ dma2Δ* cells. A–B: See legend of Figure 6 for details. C: Cycling cultures of wild type (ySP8820) and *dma1Δ dma2Δ* (ySP8821) cells expressing HA-tagged Elm1 (Elm1-HA3) were arrested in G1 by alpha factor and released into the cell cycle at 25°C. Cell samples were collected at the indicated time points for western blot analysis of Elm1-HA3 protein levels. The levels of Pgk1 were used as loading control.
(TIF)

Figure S7 Recruitment of Kcc4 to the bud neck is not affected by *DMA1* and *DMA2* deletion. A–C: Wild type (ySP8849) and *dma1Δ dma2Δ* (ySP8826) cells expressing Kcc4-eGFP were arrested in G1 by alpha factor and released into the cell cycle at 25°C (time 0). Cell samples from the untreated culture were withdrawn at the indicated times for FACS analysis of DNA contents (A) and to score budding, nuclear division and bud neck localization of Kcc4-eGFP (B). Representative micrographs were taken 60′ after release and fluorescence intensity was quantified and plotted at time 60′ in 100 cells for each strain (C). Scale bars: 5 µm.
(TIF)

Figure S8 Lack of Dma proteins partially rescues the mitotic exit delay, but not the lethality, caused by *KIN4* overexpression. A–B: Cycling cultures of untransformed wild type (W303) and of wild type (ySP7796), *bub2Δ* (ySP9559) and *dma1Δ dma2Δ* (ySP9557) strains carrying a copy of *GAL1-KIN4* integrated in the genome [25] were grown in YEPR, arrested in G1 by alpha factor at 25°C and, after 30′ induction with 2% galactose, released into the cell cycle in YEPRG (time = 0). Alpha factor (10 µg/ml) was re-added 75′ after release to arrest cells in the next G1 phase. Cell samples were withdrawn at the indicated time points to determine DNA contents by FACS analysis (A) and to score budding and spindle elongation by immunofluorescence of tubulin (B). C: Serial dilutions of the strains in (A,B) were spotted on glucose- and galactose-containing plates and incubated at 25°C for 2 days.
(TIF)

Figure S9 Lack of Dma proteins does neither affect Elm1 kinase activity nor Kin4 and Rts1 protein levels. A: Elm1-HA3 was immunoprecipitated with anti-HA antibodies from cell extracts of wild type (ySP8820) and *dma1Δ dma2Δ* (ySP8821) cells, either cycling (cyc) or arrested in the cell cycle by HU or nocodazole (noc) addition. Wild type cells expressing untagged Elm1 (W303) were used as negative control. A fraction of the total extracts used for the immunoprecipitations (input) was analysed by western blot with anti-HA and anti-Pgk1 (loading control) antibodies. Elm1 kinase assays were carried out for 30′ at 30°C to detect Elm1 autophosphorylation. An asterisk indicates an aspecific band appearing in the kinase assays. B: Cycling cultures of wild type (ySP8986 and ySP9133) and *dma1Δ dma2Δ* (ySP8987 and ySP9127) cells expressing either Kin4-HA3 or Rts1-HA3 were split in three: one aliquot was left untreated (cyc), one was treated with 150 mM HU and one with 15 µg/ml of nocodazole (noc) for 3 hours at 25°C. Crude protein extracts were analysed by western blot with anti-HA and anti-Pgk1 (loading control) antibodies.
(TIF)

Table S1 Yeast strains used in this study.
(DOC)
