# Supplementary figures and images for: Budding Yeast Dma Proteins Control Septin Dynamics and the Spindle Position Checkpoint by Promoting the Recruitment of the Elm1 Kinase to the Bud Neck
Source: PLoS Genet. 2012 Apr 26;8(4):e1002670. doi: 10.1371/journal.pgen.1002670 (PMC3343086; doi:10.1371/journal.pgen.1002670)

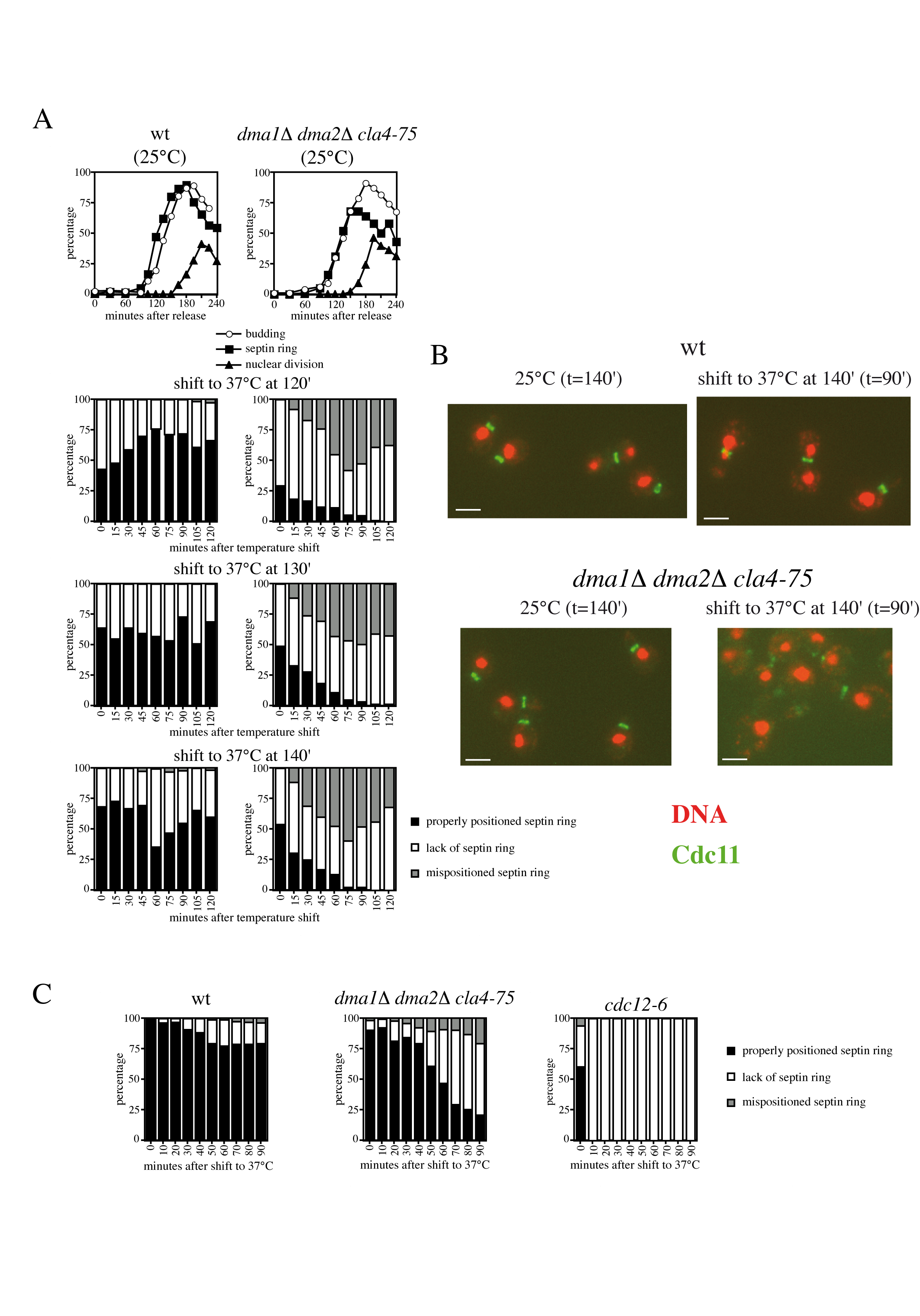

Supplement: Figure S1 — Septin ring maintenance throughout the cell cycle requires Cla4 and Dma proteins. A–B: Small unbudded cells of wild type (W303) and dma1Δ dma2Δ cla4-75 (ySP5247) strains were isolated by centrifugal elutriation and resuspended in fresh medium at the permissive temperature (25°C) at time 0 (upper graphs). Aliquots of these cultures were then shifted to 37°C after 120, 130 and 140 minutes (bottom panels). At the indicated times, cell samples were taken for FACS analysis of DNA contents (not shown), for scoring budding and nuclear division (shown only at 25°C) and for the septin ring analysis as in Figure 1B. Micrographs of representative cells are shown in (B). Scale bars: 5 µm. C: Logarithmically growing cultures of wild type (W303), dma1Δ dma2Δ cla4-75 (ySP5264) and cdc12-6 (ySP5182) cells were arrested in mitosis by 2.5 hours nocodazole treatment at 25°C and then shifted to 37°C, followed by FACS analysis of DNA contents (not shown) and in situ immunofluorescence analysis of the septin ring with anti-Cdc11 antibodies at the indicated times. We confirmed that the mitotic arrest was maintained throughout the time course in all cell cultures. (TIF) [file pgen.1002670.s001.tif]

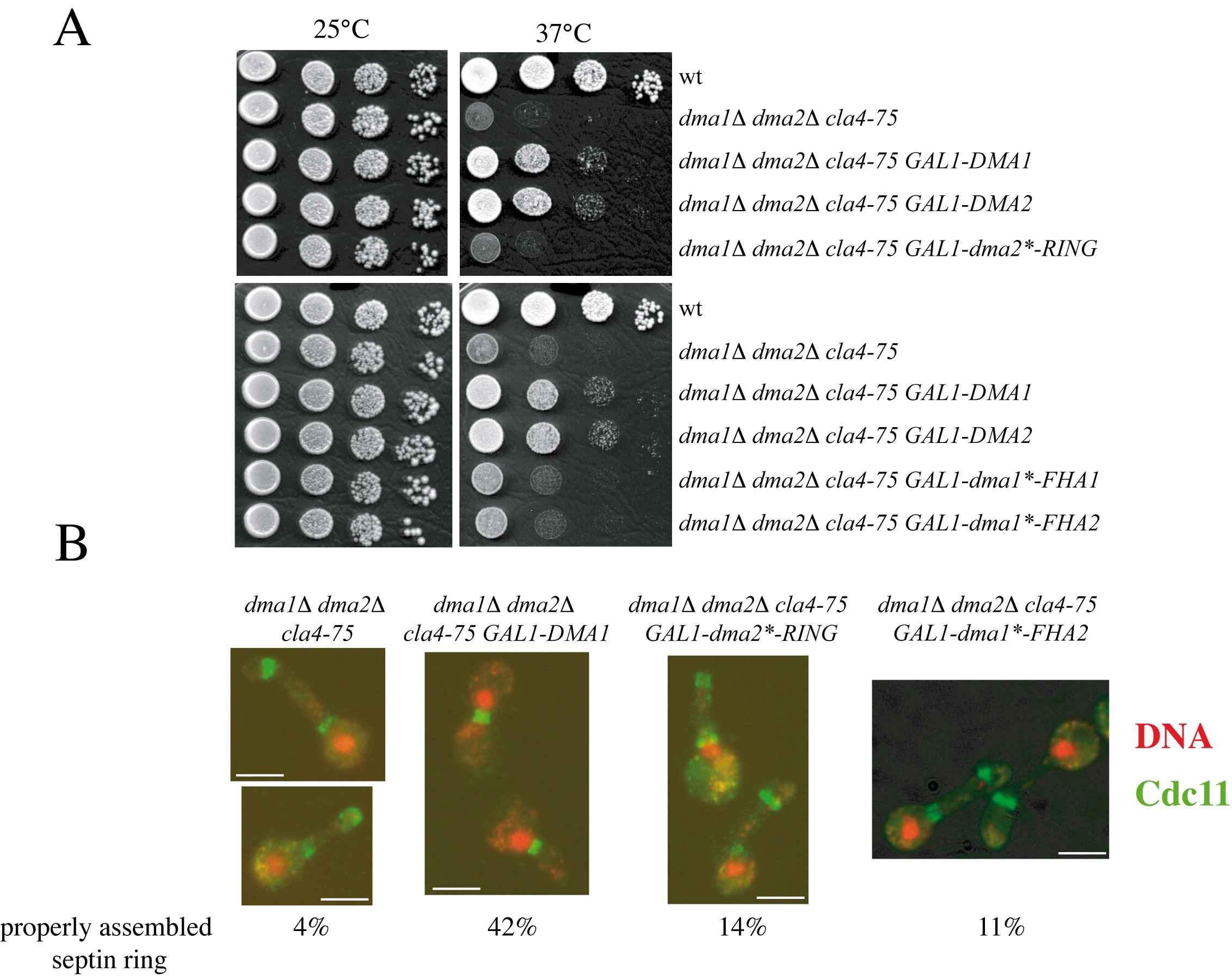

Supplement: Figure S2 — The FHA and RING domains are required for Dma proteins' function in septin ring organization. A: Serial dilutions of stationary phase cultures of the strains with the indicated genotypes were grown in synthetic raffinose medium lacking leucine at 25°C, spotted on YEPRG and incubated at the indicated temperatures for 2 days. dma1*FHA1: dma1-S220A, H223L; dma1*FHA2: dma1-G192E; dma2*RING: dma2-C451S, H456A [67]. B: Strains with the indicated genotypes were grown in synthetic medium lacking leucine at 25°C and arrested in G1 by alpha factor. 1% galactose was added 30 minutes before the release in YEPRG at 37°C. The septin ring was stained by in situ immunofluorescence with anti-Cdc11 antibodies and micrographs were taken after 3 hours at 37°C. An asterisk indicates the missense mutations in the Dma RING and FHA domains that are detailed in (A). Scale bars: 5 µm. (TIF) [file pgen.1002670.s002.tif]

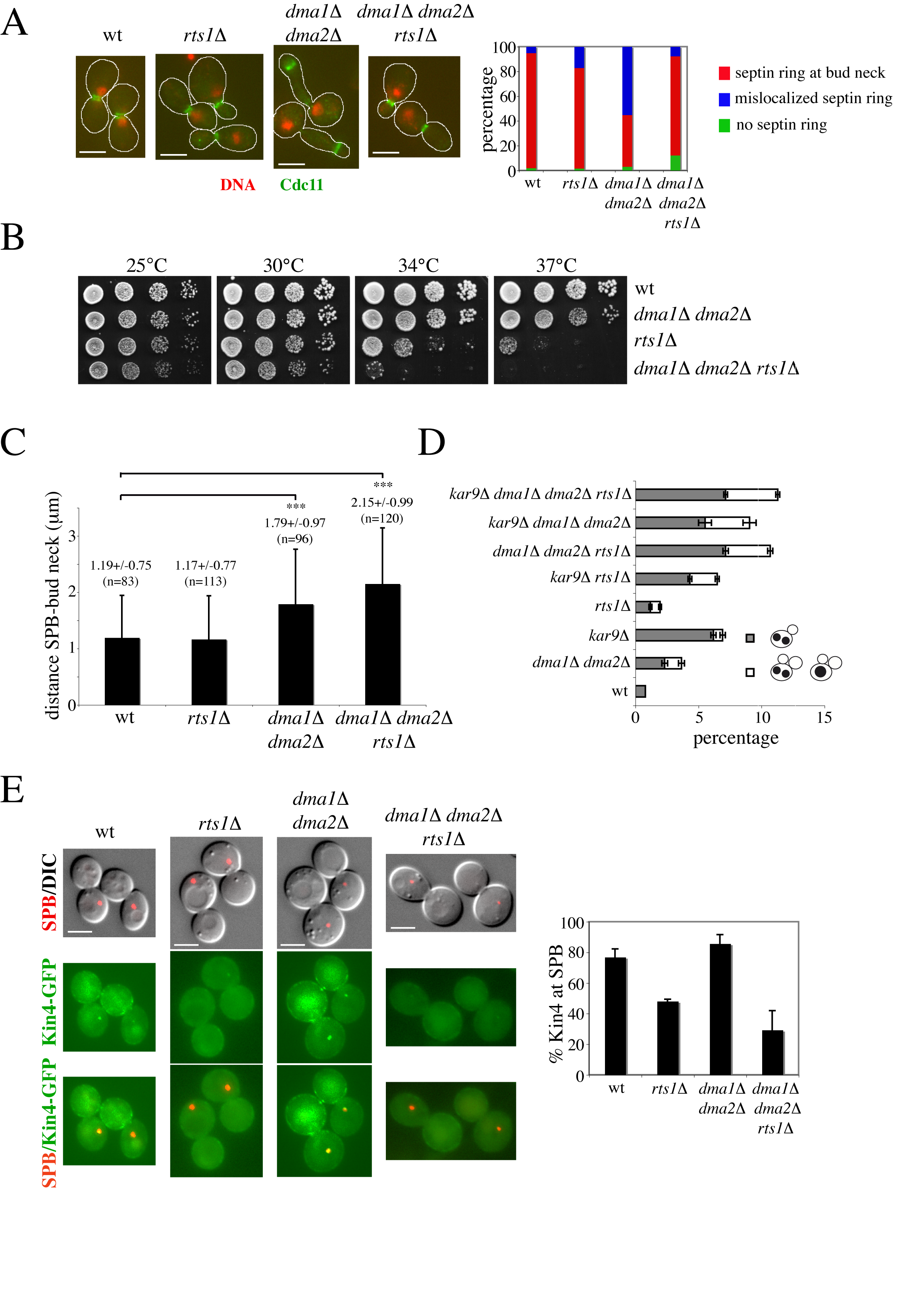

Supplement: Figure S3 — Lack of Rts1 does not suppress the spindle positioning and SPOC defects of dma1Δ dma2Δ mutants. A: Cycling cultures of wild type (W303), dma1Δ dma2Δ (ySP1569), rts1Δ (ySP9616) and dma1Δ dma2Δ rts1Δ (ySP9617) cells were treated with HU at 25°C and fixed after 6 hours to analyse the septin ring by in situ immunofluorescence with anti-Cdc11 antibodies. Scale bars: 5 µm. B: Serial dilutions of the strains in (A) were spotted on YEPD plates and incubated for 2 days at the indicated temperatures. C: Average SPB distance from the bud neck was measured as in Figure 7B in logarithmically growing cultures of wild type (ySP9594), dma1Δ dma2Δ (ySP9593), rts1Δ (ySP9596) and dma1Δ dma2Δ rts1Δ (ySP9595) cells expressing Spc42-mCherry to visualize SPBs. D: Cycling cultures of wild type (W303), dma1Δ dma2Δ (ySP1569), kar9Δ (ySP6270), rts1Δ (ySP9616), kar9Δ rts1Δ (ySP9664), dma1Δ dma2Δ rts1Δ (ySP9662), kar9Δ dma1Δ dma2Δ (ySP9661) and kar9Δ dma1Δ dma2Δ rts1Δ (ySP9663) were shifted to 37°C for 3 hours, followed by scoring cell morphology and nuclear division after nuclear staining with propidium iodide. Histograms represent average values of three independent experiments, with bars showing standard errors. At least 500 cells were scored in each experiment. E: Wild type (ySP8996), rts1Δ (ySP9467) dma1Δ dma2Δ (ySP8994) and dma1Δ dma2Δ rts1Δ (ySP9466) cells expressing Kin4-GFP and Spc42-mCherry were arrested in mitosis with nocodazole. Z-projected images of representative cells with Kin4-GFP co-localizing with Spc42-mCherry are shown. The histograms on the right side show the quantification of data with standard error bars from three independent repeats. At least 100 cells were scored for each strain in each experiment. Note that in rts1Δ strains Kin4-GFP signals at SPBs, when present, are always very faint. Scale bars: 5 µm. (TIF) [file pgen.1002670.s003.tif]

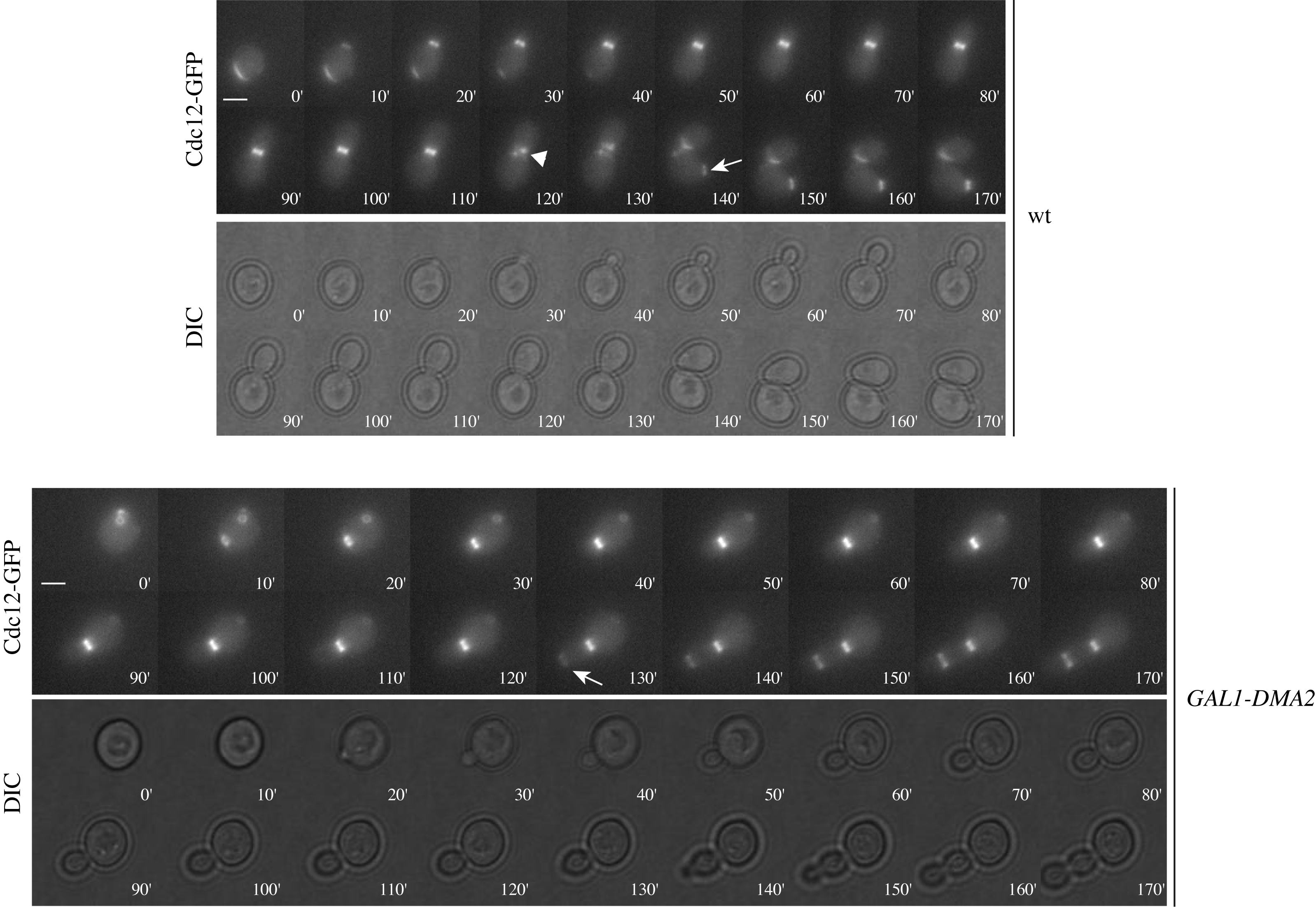

Supplement: Figure S4 — DMA2 overexpression delays septin ring splitting and disassembly. Wild type (upper rows) and GAL1-DMA2 cells (lower rows) expressing Cdc12-GFP were recorded every 10 minutes by time lapse video microscopy. The arrowhead indicates septin ring splitting; the arrow indicates appearance of a new septin ring. Scale bars: 5 µm. (TIF) [file pgen.1002670.s004.tif]

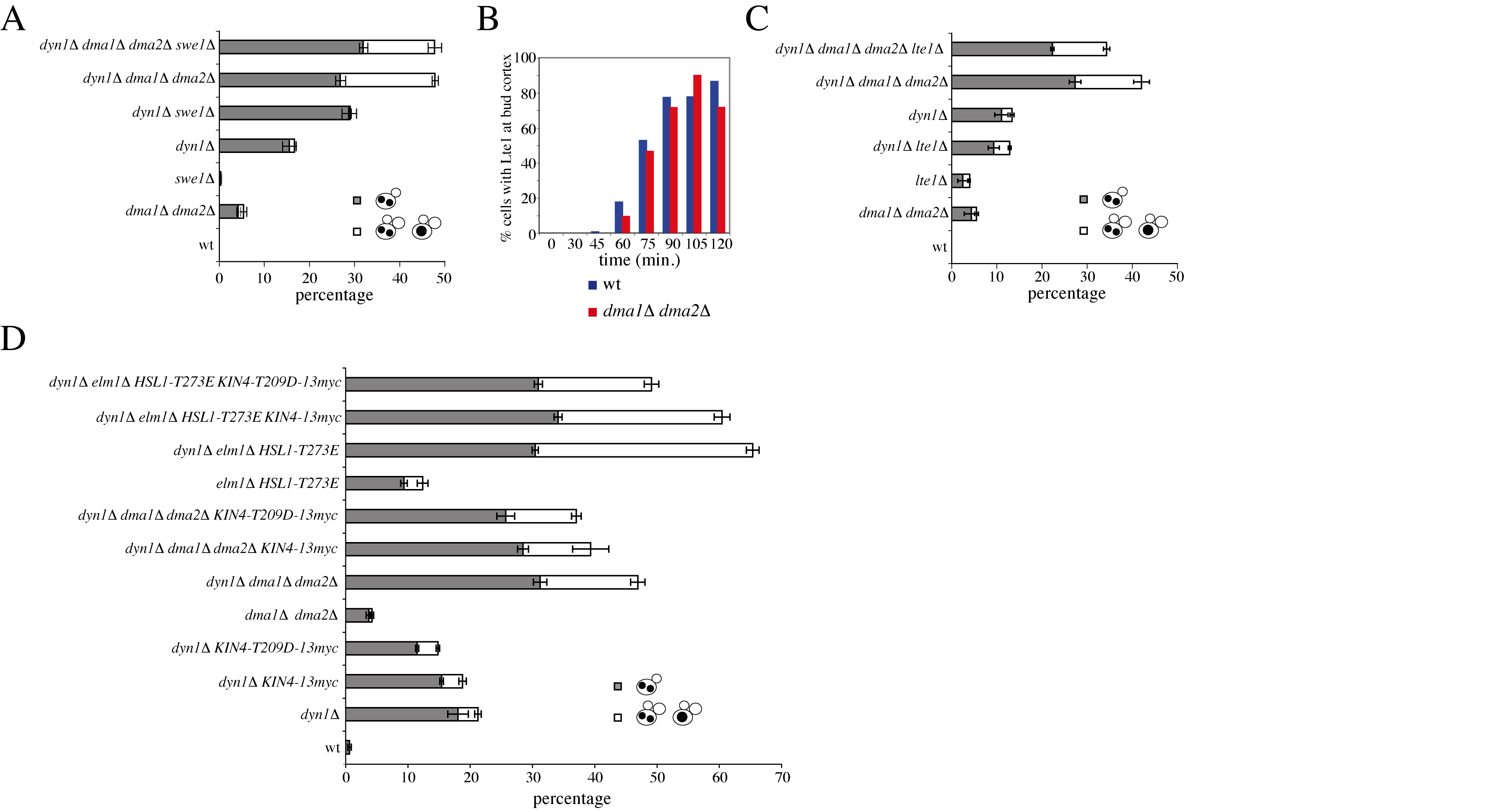

Supplement: Figure S5 — Lack of Swe1 or Lte1 does not suppress the SPOC defect of dma1Δ dma2Δ mutants. A: Exponentially growing cultures of wild type (W303), dma1Δ dma2Δ (ySP1569), swe1Δ (ySP1370), dyn1Δ (ySP6292), dyn1Δ swe1Δ (yRF1301), dyn1Δ dma1Δ dma2Δ (ySP7454) and dyn1Δ dma1Δ dma2Δ swe1Δ (yRF1306) cells were shifted to 14°C for 16 hours, followed by scoring cell morphology and nuclear division after nuclear staining with propidium iodide. Histograms represent average values of three independent experiments, with bars showing standard errors. At least 500 cells were scored in each experiment. B: Cycling cultures of wild type (ySP3333) and dma1Δ dma2Δ (ySP4380) strains carrying a LTE1-GFP construct under the control of the galactose-inducible GAL1 promoter were grown in YEPR, arrested in G1 with a factor, pre-induced with 1% galactose for 30 minutes and finally released in YEPRG at 25°C (time = 0). Cells were collected at the indicated time points for FACS analysis of DNA contents (not shown) and localization of Lte1-GFP at the bud cortex (graph). At least 200 cells were scored at each time point. C: Exponentially growing cultures of wild type (W303), dma1Δ dma2Δ (ySP1569), lte1Δ (ySP8657), dyn1Δ lte1Δ (yRF1434), dyn1Δ (ySP6292), dyn1Δ dma1Δ dma2Δ (ySP7454) and dyn1Δ dma1Δ dma2Δ lte1Δ (ySP9523) cells were treated and scored as in (A). D: Cycling cultures of wild type (W303), dyn1Δ (ySP6292), dyn1Δ KIN4-13myc (ySP9238), dyn1Δ KIN4-T209D-13myc (ySP9239), dma1Δ dma2Δ (ySP1569), dyn1Δ dma1Δ dma2Δ (ySP7454), dyn1Δ dma1Δ dma2Δ KIN4-13myc (ySP9236), dyn1Δ dma1Δ dma2Δ KIN4-T209D-13 myc (ySP9240), elm1Δ HSL1-T273E (ySP9214), dyn1Δ elm1Δ HSL1-T273E (ySP9244), dyn1Δ elm1Δ HSL1-T273E KIN4-13myc (ySP9289) and dyn1Δ elm1Δ HSL1-T273E KIN4-T209D-13myc (ySP9243) cells were treated and scored as in (A). (TIF) [file pgen.1002670.s005.tif]

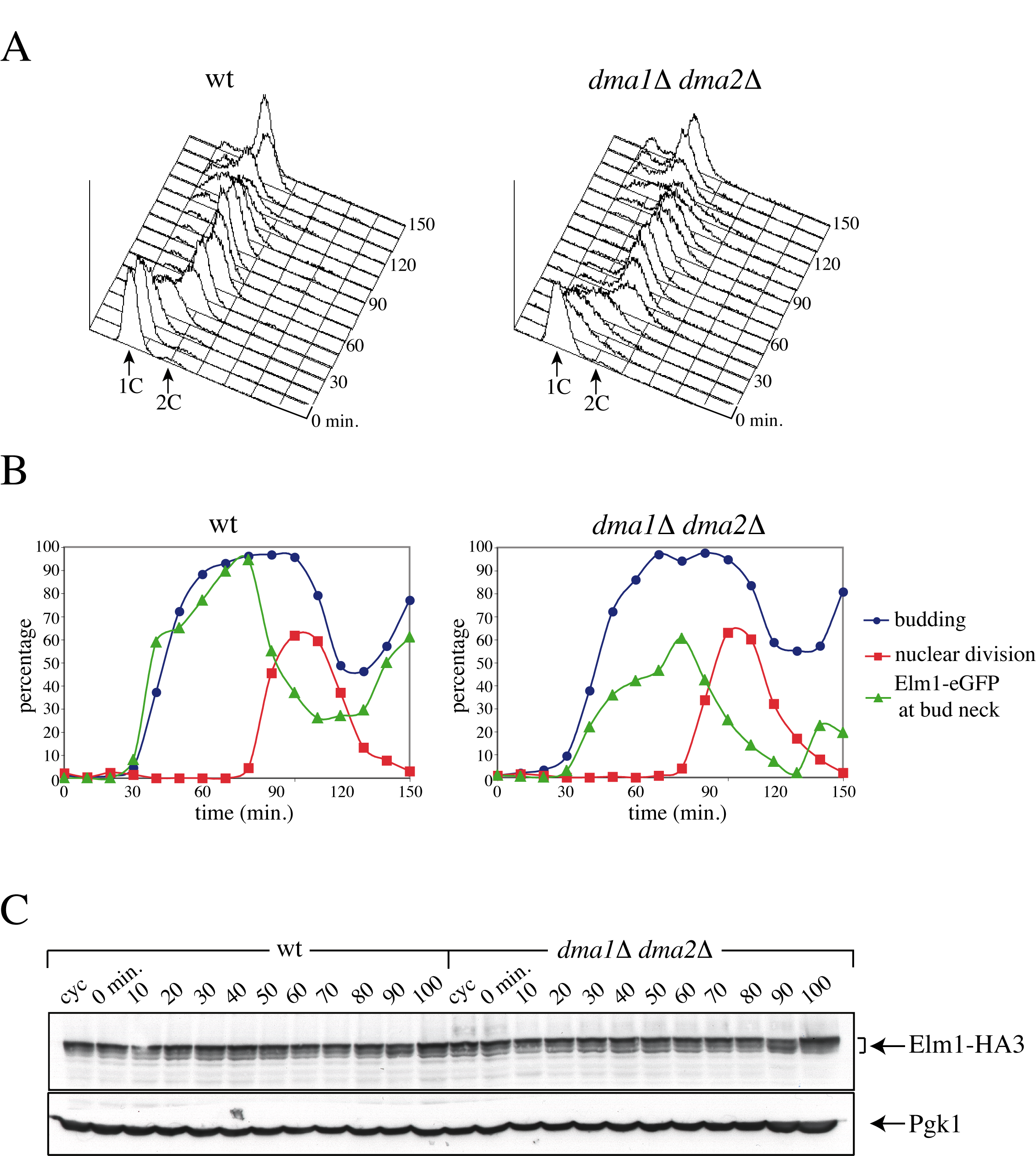

Supplement: Figure S6 — Elm1 localization and protein levels during the cell cycle in wild type and dma1Δ dma2Δ cells. A–B: See legend of Figure 6 for details. C: Cycling cultures of wild type (ySP8820) and dma1Δ dma2Δ (ySP8821) cells expressing HA-tagged Elm1 (Elm1-HA3) were arrested in G1 by alpha factor and released into the cell cycle at 25°C. Cell samples were collected at the indicated time points for western blot analysis of Elm1-HA3 protein levels. The levels of Pgk1 were used as loading control. (TIF) [file pgen.1002670.s006.tif]

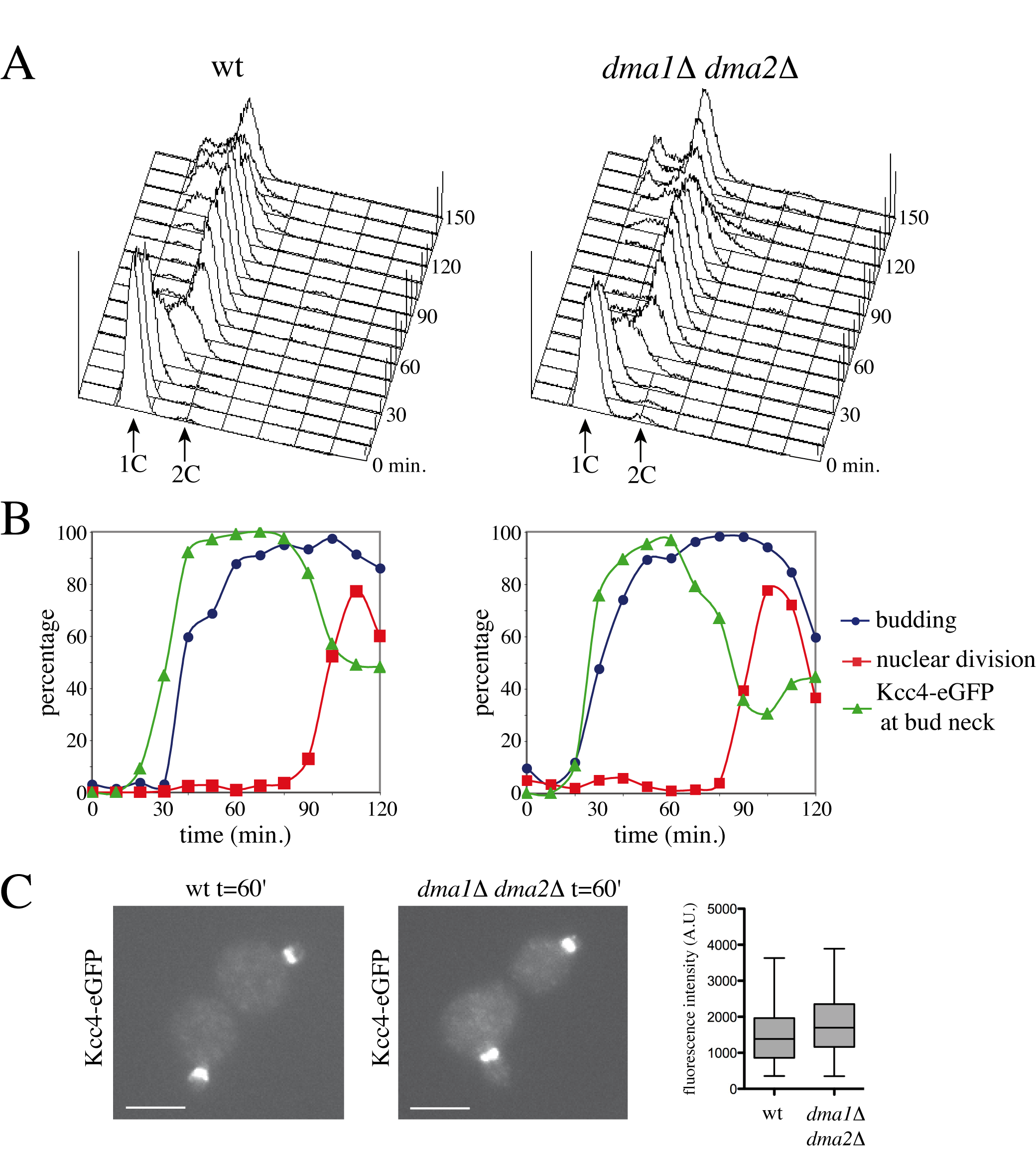

Supplement: Figure S7 — Recruitment of Kcc4 to the bud neck is not affected by DMA1 and DMA2 deletion. A–C: Wild type (ySP8849) and dma1Δ dma2Δ (ySP8826) cells expressing Kcc4-eGFP were arrested in G1 by alpha factor and released into the cell cycle at 25°C (time 0). Cell samples from the untreated culture were withdrawn at the indicated times for FACS analysis of DNA contents (A) and to score budding, nuclear division and bud neck localization of Kcc4-eGFP (B). Representative micrographs were taken 60′ after release and fluorescence intensity was quantified and plotted at time 60′ in 100 cells for each strain (C). Scale bars: 5 µm. (TIF) [file pgen.1002670.s007.tif]

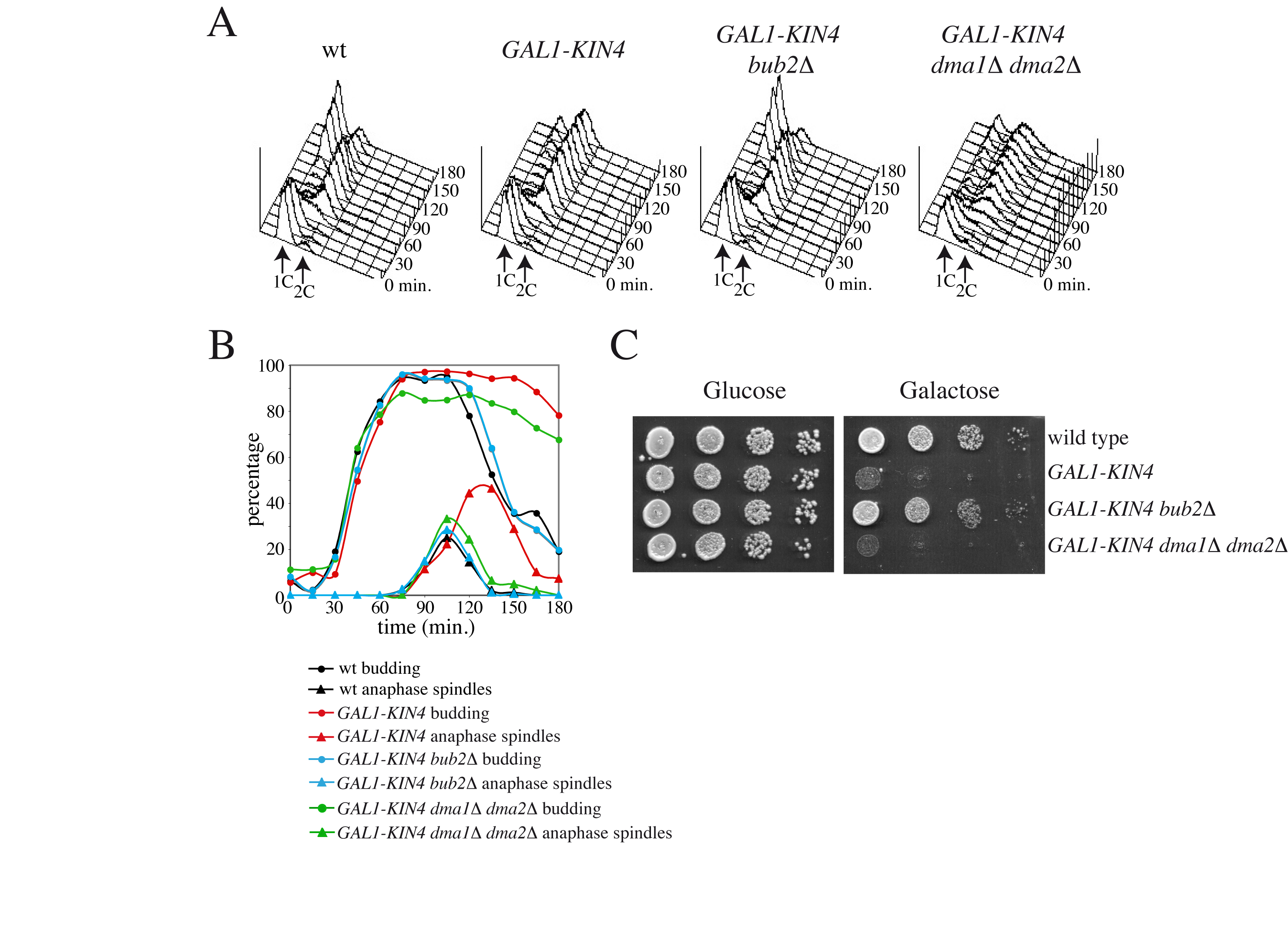

Supplement: Figure S8 — Lack of Dma proteins partially rescues the mitotic exit delay, but not the lethality, caused by KIN4 overexpression. A–B: Cycling cultures of untransformed wild type (W303) and of wild type (ySP7796), bub2Δ (ySP9559) and dma1Δ dma2Δ (ySP9557) strains carrying a copy of GAL1-KIN4 integrated in the genome [25] were grown in YEPR, arrested in G1 by alpha factor at 25°C and, after 30′ induction with 2% galactose, released into the cell cycle in YEPRG (time = 0). Alpha factor (10 µg/ml) was re-added 75′ after release to arrest cells in the next G1 phase. Cell samples were withdrawn at the indicated time points to determine DNA contents by FACS analysis (A) and to score budding and spindle elongation by immunofluorescence of tubulin (B). C: Serial dilutions of the strains in (A,B) were spotted on glucose- and galactose-containing plates and incubated at 25°C for 2 days. (TIF) [file pgen.1002670.s008.tif]

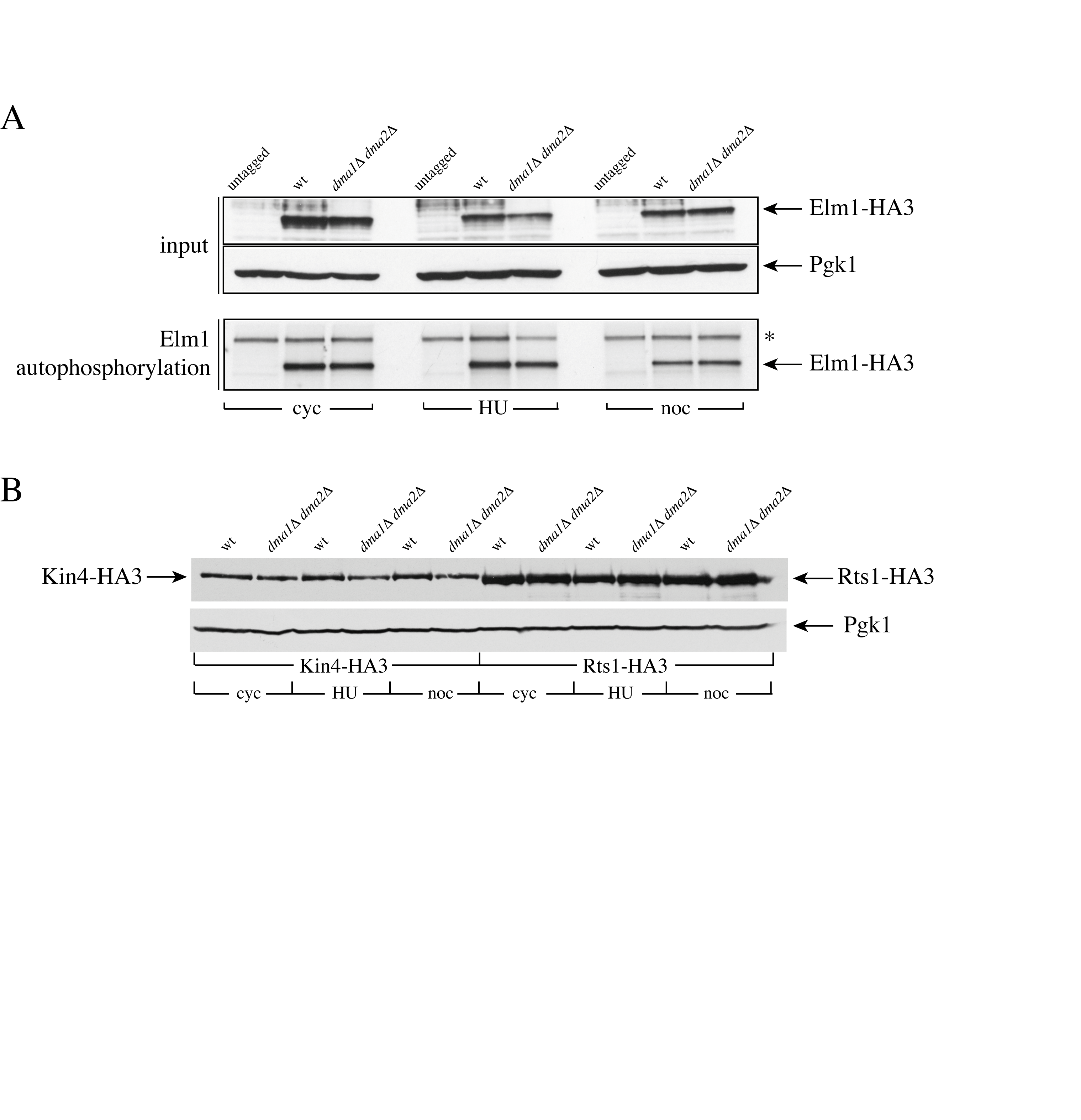

Supplement: Figure S9 — Lack of Dma proteins does neither affect Elm1 kinase activity nor Kin4 and Rts1 protein levels. A: Elm1-HA3 was immunoprecipitated with anti-HA antibodies from cell extracts of wild type (ySP8820) and dma1Δ dma2Δ (ySP8821) cells, either cycling (cyc) or arrested in the cell cycle by HU or nocodazole (noc) addition. Wild type cells expressing untagged Elm1 (W303) were used as negative control. A fraction of the total extracts used for the immunoprecipitations (input) was analysed by western blot with anti-HA and anti-Pgk1 (loading control) antibodies. Elm1 kinase assays were carried out for 30′ at 30°C to detect Elm1 autophosphorylation. An asterisk indicates an aspecific band appearing in the kinase assays. B: Cycling cultures of wild type (ySP8986 and ySP9133) and dma1Δ dma2Δ (ySP8987 and ySP9127) cells expressing either Kin4-HA3 or Rts1-HA3 were split in three: one aliquot was left untreated (cyc), one was treated with 150 mM HU and one with 15 µg/ml of nocodazole (noc) for 3 hours at 25°C. Crude protein extracts were analysed by western blot with anti-HA and anti-Pgk1 (loading control) antibodies. (TIF) [file pgen.1002670.s009.tif]
