# Supplementary material for: Budding Yeast Dma Proteins Control Septin Dynamics and the Spindle Position Checkpoint by Promoting the Recruitment of the Elm1 Kinase to the Bud Neck
Source: PLoS Genet. 2012 Apr 26;8(4):e1002670. doi: 10.1371/journal.pgen.1002670 (PMC3343086; doi:10.1371/journal.pgen.1002670)
Supplement: Table S1 — Yeast strains used in this study. (DOC) [file pgen.1002670.s010.doc]

**Table S1. List of *Saccharomyces cerevisiae* strains used in this study**

**Name** **Relevant genotype**

ySP294 *MATa, cdc12-1*

ySP1370 *MATa,* *swe1::LEU2*

ySP1569 *MATa, dma1:: KlTRP1, dma2::KlLEU2*

ySP3018 *MATa, ura3::4X URA3::GAL1-DMA2*

ySP3070 *MATa, [CEN-URA3-CDC3-GFP]*

ySP3333 *MATa, GAL1-GFP-LTE1::KanMX4,*

ySP4319 *MATa, dma1::KlTRP1, dma2::KlLEU2, [CEN-URA3-CDC3-GFP]*

ySP4320 *MATa, cla4::KanMX4, [CEN-TRP1-cla4-75], [CEN-URA3-CDC3-GFP]*

ySP4321 *MATa, dma1::KlTRP1, dma2::KlLEU2, cla4::KanMX4, [CEN-TRP1-cla4-75], [CEN-URA3-CDC3-GFP]*

ySP4380 *MATa dma1::KlTRP1, dma2::KlLEU2, GAL1-GFP-LTE1::KanMX4*

ySP5182 *MATalpha, cdc12-6*

ySP5244 *MATa, ura3::4X URA3::GAL1-DMA2, cdc12-1*

ySP5247 *MATa, dma1::KlTRP1, dma2::HPHMX, cla4::KanMX4,* *[CEN-URA3-cla4-75]*

ySP5264 *MATalpha, dma1::KlTRP1, dma2::HPHMX, cla4::KanMX4, [CEN-URA3-cla4-75]*

ySP6238 *MATa, dma1::KlTRP1, dma2::HPHMX, cla4::KanMX4, [CEN-URA3-cla4-75], swe1::LEU2*

ySP6270 *MATa, kar9::KanMX4*

ySP6292 *MATa, dyn1::KanMX4*

ySP7342 *MATa, shs1::natNT2*

ySP7454 *MATa, dyn1::KanMX4, dma1::KlTRP1, dma2::KlLEU2*

ySP7554 *MATalpha, ura3::4X URA3::GAL1-DMA2, shs1::natNT2*

ySP7555 *MATa, ura3::4X URA3::GAL1-DMA2, shs1::natNT2*

ySP7672 *MATa, dma1::KlTRP1, dma2::HPHMX, cla4::KanMX4, swe1::LEU2, [CEN-URA3-cla4-75] , lte1::SpHIS5*

ySP7681 *MATa, dma1::KlTRP1, dma2::HPHMX, cla4::KanMX4, [CEN-LEU2-GAL1-DMA2-2HA], [CEN-URA3-cla4-75]*

ySP7682 *MATa, dma1::KlTRP1, dma2::HPHMX, cla4::KanMX4, [CEN-LEU2-GAL1-DMA1-2HA], [CEN-URA3-cla4-75]*

ySP7684 *MATa, dma1::KlTRP1, dma2::HPHMX, cla4::KanMX4, [CEN-LEU2-GAL1-DMA2- C451S,H456A], [CEN-URA3-cla4-75]*

ySP7685 *MATa, dma1::KlTRP1, dma2::HPHMX, cla4::KanMX4, [CEN-LEU2-GAL1-DMA1- G192E-2HA], [CEN-URA3-cla4-75]*

ySP7686 *MATa, dma1::KlTRP1, dma2::HPHMX, cla4::KanMX4, [CEN-LEU2-GAL1-DMA1- S220A, H223L-HA], [CEN-URA3-cla4-75]*

ySP7766 *MATa, dma1::KlTRP1, dma2::HPHMX, cla4::KanMX4, [CEN-URA3-cla4-75], GAL1-GFP-LTE1::KanMX4*

ySP7771 *MATa, dma1::KlTRP1, dma2::HPHMX, cla4::KanMX4, [CEN-URA3-cla4-75], swe1::LEU2, BUB2-HA3::KlTRP1*

ySP7789 *MATa, dma1::KlTRP1, dma2::HPHMX, cla4::KanMX4, [CEN-URA3-cla4-75], swe1::LEU2, TEM1-HA3::KlURA3*

ySP7796 *MATa, trp1::GAL-KIN4, CDC14-3HA*

ySP7819 *MATa, dma1::KlTRP1, dma2::HPHMX, cla4::KanMX4, [CEN-URA3-cla4-75], swe1::LEU2, BFA1-HA6::HIS3MX6*

ySP8173 *MATa, dma1::KlTRP1, dma2::KlLEU2, GFP-CDC12::URA3*

ySP8176 *MATa, GFP-CDC12::URA3*

ySP8193 *MATa, dma1::KlTRP1, dma2::KlLEU2, cla4::KanMX4, [CEN-TRP1-cla4-75]GFP-CDC12::URA3*

ySP8280 *MATa, cla4::KanMX4, GFP-CDC12::URA3*

ySP8296 *MATa, cla4::KanMX4, [CEN-TRP1-cla4-75], GFP-CDC12::URA3*

ySP8657 *MATalpha, lte1:: SpHIS5*

ySP8679 *MATa, dma1::KlTRP1, dma2::HPHMX, cla4::KanMX4, rts1::KanMX6*

ySP8634 *MATalpha, ura3::4X URA3::GAL1-DMA2, GFP-CDC12::URA3*

ySP8701 *MATa, rts1::kanMX6, GFP-CDC12::URA3*

ySP8703 *MATalpha, dma1::KlTRP1, dma2::KlLEU2, rts1::kanMX4, GFP-CDC12::URA3*

ySP8813 *MATa, ELM1-eGFP::kanMX4*

ySP8820 *MATa, ELM1-HA3::KIURA3*

ySP8821 *MATa, ELM1-HA3::KIURA3, dma1::KlTRP1, dma2::KlLEU2*

ySP8826 *MATa, dma1::KlTRP1, dma2::KlLEU2, KCC4-eGFP::kanMX4*

ySP8829 *MATa, dma1::KlTRP1, dma2::KlLEU2, ELM1-eGFP::kanMX4*

ySP8849 *MATa, KCC4-eGFP::kanMX4*

ySP8986 *MATa, KIN4-HA3::KlURA3*

ySP8987 *MATa, dma1::KlTRP1, dma2::KlLEU2, KIN4-HA3::KlURA3*

ySP8994 *MATa, dma1::KlTRP1, dma2::KlLEU2, KIN4-GFP::HIS3MX6, SPC42-mCherry::natNT2*

ySP8995 *MATalpha, KIN4-GFP::HIS3MX6, SPC42-mCherry::natNT2*

ySP8996 *MATa, KIN4-GFP::HIS3MX6, SPC42-mCherry::natNT2*

ySP8997 *MATa, elm1::natNT2, KIN4-HA3::KlURA3*

ySP9127 *MATa, dma1::KlTRP1, dma2::KlLEU2, RTS1-HA3::URA3*

ySP9133 *MATa, RTS1-HA3::URA3*

ySP9214 *MATalpha, elm1::natNT2, trp1::TRP1::HSL1(T273E)-13myc*

ySP9236 *MATa, dma1::KlTRP1, dma2::KlLEU2, dyn1::KanMX4, KIN4-13myc::KanMX4*

ySP9238 *MATa, dyn1::KanMX4, KIN4-13myc::KanMX4*

ySP9239 *MATalpha, dyn1::KanMX4, KIN4-T209D-13myc::KanMX4*

ySP9240 *MATalpha, dma1::KlTRP1, dma2::KlLEU2, dyn1::KanMX4, KIN4-T209D-13myc::KanMX4*

ySP9243 *MATa, dyn1::KanMX4, elm1::natNT2, trp1::TRP1::HSL1(T273E)-13myc, KIN4-T209D-13myc::KanMX4*

ySP9244 *MATa, dyn1::KanMX4, elm1::natNT2, trp1::TRP1::HSL1(T273E)-13myc*

ySP9289 *MATalpha, dyn1::KanMX4, elm1::natNT2, trp1::TRP1::HSL1(T273E)-13myc, KIN4-13myc::KanMX4*

ySP9466 *MATalpha, dma1::KlTRP1, dma2::KlLEU2, rts1::kanMX6, KIN4-GFP::HIS3MX6, SPC42-mCherry::natNT2*

ySP9467 *MATalpha, rts1::kanMX6, KIN4-GFP::HIS3MX6, SPC42-mCherry::natNT2*

ySP9476 *MATa, cla4::kanMX4, rts1::kanMX6, GFP-CDC12::URA3*

ySP9484 *MATalpha, dma1::KlTRP1, dma2::KlLEU2, rts1::kanMX6, GFP-CDC12::URA3*

ySP9485 *MATalpha, rts1::kanMX6, KIN4-HA3::KlURA3*

ySP9487 *MATa, dma1::KlTRP1, dma2::KlLEU2, BNI4-∆420-tdimer2::KanMX6, KIN4-HA3::KlURA3*

ySP9489 *MATa, elm1::HPHMX, BNI4-∆420-tdimer2::KanMX6, KIN4-HA3::KlURA3*

ySP9492 *MATa, dma1::KlTRP1, dma2::KlLEU2, dyn1::KanMX4, BFA1-eGFP::KanMX4, ura3::URA3::mCherry-Tub1*

ySP9493 *MATa, dyn1::KanMX4, BFA1-eGFP::KanMX4, ura3::URA3::mCherry-Tub1*

ySP9523 *MATa, dma1::KlTRP1, dma2::KlLEU2, dyn1::KanMX4, lte1::SpHIS5*

ySP9557 *MATa, trp1::TRP1::GAL-KIN4, CDC14-3HA, dma1::KlLEU2, dma2::HPHMx*

ySP9559 *MATa, trp1::TRP1::GAL-KIN4, CDC14-3HA, bub2::HIS3*

ySP9587 *MATalpha, dma1::KlTRP1, dma2::KlLEU2, BNI4-elm1∆420-tdimer2::KanMX6*

ySP9588 *MATa, dma1::KlTRP1, dma2::KlLEU2, elm1::HPHMX, BNI4-elm1∆420-tdimer2::KanMX6*

ySP9589 *MATalpha, BNI4-elm1∆420-tdimer2::KanMX6*

ySP9590 *MATalpha, BNI4-elm1∆420-tdimer2::KanMX6, dyn1::KanMX4*

ySP9592 *MATa, dma1::KlTRP1, dma2::KlLEU2, dyn1::KanMX4, BNI4-elm1∆420-tdimer2::KanMX6*

ySP9593 *MATa, dma1::KlTRP1, dma2::KlLEU2, SPC42-mCherry::natNT2*

ySP9594 *MATalpha, SPC42-mCherry::natNT2*

ySP9595 *MATa, dma1::KlTRP1, dma2::KlLEU2, rts1::kanMX6, SPC42-mCherry::natNT2*

ySP9596 *MATa, rts1::kanMX4, SPC42-mCherry::natNT2*

ySP9599 *MATa, elm1::HPHMX, BNI4-elm1∆420-tdimer2::KanMX6*

ySP9616 *MATalpha, rts1:: natNT2*

ySP9617 *MATa, dma1::KlTRP1, dma2::KlLEU2, rts1::natNT2*

ySP9634 *MATa, dma1::KlTRP1, dma2::KlLEU2, BNI4-elm1∆420-tdimer2::KanMX6, SPC42-mCherry::natNT2*

ySP9636 *MATa, BNI4-elm1∆420-tdimer2::KanMX6, SPC42-mCherry::natNT2*

ySP9661 *MATalpha, dma1::KlTRP, dma2::HPHMX, kar9::KanMX4*

ySP9662 *MATa, dma1::KlTRP, dma2::HPHMX, rts1::natNT2*

ySP9663 *MATa, dma1::KlTRP, dma2::HPHMx, rts1::natNT2, kar9::KanMX4*

ySP9664 *MATalpha, rts1::natNT2, kar9::KanMX4*

yRF1301 *MATa, dyn1::KanMX4, swe1::LEU2*

yRF1306 *MATa, dyn1::KanMX4, dma1::KlTRP1, dma2::KlLEU2, swe1::LEU2*

yRF1434 *MATa, dyn1::KanMX4, lte1::SpHIS5*

Plasmids are indicated in brackets
